# Efficacy and safety of antibody-drug conjugates in the treatment of advanced urological cancers: a systematic review and a meta-analysis

**DOI:** 10.3389/fphar.2025.1583654

**Published:** 2025-09-01

**Authors:** Tian Liu, Xiao Xie, Yangz-Zi Ren, Zongyu Li, Maoping Cai, Yuzhong Yu, Lin Zhuo

**Affiliations:** ^1^ Department of Urology, Pingxiang Affiliated Hospital, Pingxiang, China; ^2^ Department of Oncology, The First Affiliated Hospital of Guangzhou University of Chinese Medicine, Guangzhou, Guangdong, China; ^3^ Department of Urology, Tongji Hospital, Tongji Medical College, Huazhong University of Science and Technology, Wuhan, China; ^4^ Department of Urology, Fudan University Shanghai Cancer Center, Shanghai, China; ^5^ Department of Urology, Nanfang Hospital, Southern Medical University, Guangzhou, Guangdong, China

**Keywords:** antibody-drug conjugates, urological cancers, efficacy, safety, meta-analysis

## Abstract

**Background:**

Antibody-drug conjugates (ADCs) offer novel therapeutic options for advanced urological cancers, but their efficacy and safety vary across cancer types. Many non-urothelial cancer ADC trials are small, nonrandomized studies with limited validated evaluation indicators. The purpose of this study is to evaluate the efficacy and safety of ADCs across various urological cancers.

**Methods:**

Relevant studies were identified through searches in Embase, PubMed, Web of Science, Cochrane Library, CNKI, VIP database and WanFang dataset, including randomized controlled trials, single-arm studies, and retrospective analyses on ADCs for advanced urological cancers. RoB 2.0, MINORS, and NOS were used for quality assessment, with R 4.4.0 for data analysis.

**Results:**

This meta-analysis included 46 studies with 3,250 patients, covering urothelial cancer (29 studies), renal cell carcinoma (5 studies), testicular cancer (2 studies), and metastatic castration-resistant prostate cancer (10 studies). Three ADCs for urothelial cancer have received approval, including enfortumab vedotin (EV), sacituzumab govitecan (SG), and the HER2-ADC vedicilizumab (RC-48)/disitamab vedotin (DV). For urothelial cancer, the pooled overall response rate (ORR) was 43% (95% CI: 39%–47%) and disease control rate (DCR) was 76% (95% CI: 71%–80%). Median overall survival (OS) and progression-free survival (PFS) were 11.55 months (95% CI: 10.63–12.47) and 5.52 months (95% CI: 5.32–5.72) for enfortumab vedotin (EV), and 15.30 months (95% CI: 11.21–19.40) and 5.80 months (95% CI: 4.88–6.72) for DV. DV combined with immunotherapy achieved a pooled median PFS of 9.78 months (95% CI: 7.73–11.83). For renal cell carcinoma, the ORR was 6% (95% CI: 2%–10%) with median OS of 12.71 months (95% CI: 9.67–15.75). For metastatic castration-resistant prostate cancer, ADC efficacy was higher in chemotherapy-experienced patients (ORR: 17% vs. 5%).

**Conclusion:**

ADCs demonstrate efficacy and safety in treating urological cancers, but further clinical trials are needed, particularly for renal, testicular, and prostate cancers, to support personalized treatment strategies.

## 1 Introduction

Globally, urological cancer accounting for 13% in all cancers has led to substantial public health burden worldwide, especially in aging societies (Bray et al., 2024; Dy et al., 2017). Urological cancers primarily comprise bladder, prostate, kidney, and testicular cancers (Bray et al., 2024). Bladder cancer was the fourth most frequent disease in 2023, with a significant rate of mortality and recurrence, according to cancer statistics. The most common disease and the second leading cause of death for men is prostate cancer (Siegel et al., 2023). As reported in World Cancer Research Fund International, kidney and testicular cancer were ranked as the 14th and 20th among all cancers, with approximately 430,000 and 74,500 new cases in 2020 worldwide (World Cancer Research Fund International, 2023). Nowadays, urological cancers have been a long-standing problem that requires more new treatments to be offered in the future.

Antibody-drug conjugates (ADCs) are designed to maximize cancer cell death while reducing cytotoxicity towards non-cancer cells and are emerging as a more promising option for targeted cancer therapies (Gabison et al., 2024). ADCs have been recognized as “biological missiles,” “Trojan horses,” and “smart chemotherapies” to a certain degree, consisting of major three components, the payload drug, the monoclonal antibody, and the chemical linker (Colombo et al., 2024). Payload drugs, known as the ‘magic bullet’ with their high cytotoxicity, are responsible for causing cancer cell death. The main compounds are monomethyl auristatin E (MMAE), nomethoxycyaline F (MMAF), and maytansinoids (DM1 and DM4). The monoclonal antibody (mAb) as the navigation system could specifically recognize and bind to specific antigens on the outer layer of cancer cells to minimize the cytotoxicity towards noncancerous cells. The chemical linker handles the combination of payloads and monoclonal antibodies (Marks and Naidoo, 2022; McCombs and Owen, 2015).

Eleven of the more than 370 new ADCs that have made it into clinical trials so far have received approval from the US Food and Drug Administration (Tarantino et al., 2022; Drago et al., 2021; Maecker et al., 2023). Numerous ADCs have been used in phase I, II, and III clinical trials for urological cancers (de Vries et al., 2023; Kollmannsberger et al., 2021; Koshkin et al., 2022; Milowsky et al., 2016; Powles et al., 2024a). In particular, three ADCs for urothelial cancer have received approval. They are enfortumab vedotin (EV), sacituzumab govitecan (SG), and the HER2-ADC vedicilizumab (RC-48). The first 2 pharmacological agents were granted approval by the FDA (U.S.Food and administration, 2021; Chang et al., 2021), whereas RC-48 is the drug that China has authorized and cleared for the treatment of urothelial cancer (Sheng et al., 2021). In the EV-302 Phase III clinical trial, the EV plus pembrolizumab group achieved an overall response rate (ORR) of 67.7% (95% CI: 63.1%–72.1%) and a disease control rate (DCR) of 86.5% in patients, more than double that of the chemotherapy group (Powles et al., 2024a). In the EV-103 study, the ORR in EV group was 45.2% (33.5%–57.3%) (O'Donnell et al., 2023). Other single-arm studies have reported variations in survival duration and tumor response rates for enfortumab vedotin (EV) in the treatment of urothelial cancer. The ORR ranges from 25% to 56% in the 1.25 mg/kg EV group (Takahashi et al., 2020; Yu et al., 2021). According to data from many studies, enfortumab vedotin (EV) may present safety risks because of the 55% (Yu et al., 2021) rate of grade 3 or worse treatment-related side events and the 44.8% prevalence of peripheral sensory neuropathy (Powles et al., 2021) in the general population. Meanwhile, in the SG clinical trials, the ORR ranged from 28.9% to 32% (Pet et al., 2024; Bardia et al., 2021). Similarly, in the RC-48 clinical trials, the ORR varied from 26.3% to 50% (Xu et al., 2022; Xu et al., 2023) demonstrating that it is necessary to pool all relevant studies to verify the efficacy and safety of ADCs in urothelial cancer. Many clinical trials in phases I and II have been conducted in recent years, despite the fact that the Food and Drug Administration has not approved any pharmaceutical drugs for the treatment of prostate, renal, or testicular cancers. Despite these advancements, the efficacy and safety of ADCs vary across different types of urological cancers. Many clinical trials for non-urothelial cancers are small, non-randomized studies with limited validated evaluation indicators. Therefore, a comprehensive systematic review and meta-analysis is needed to evaluate the overall efficacy and safety of ADCs across various urological cancers. This study aims to compile and analyze all relevant studies, including randomized controlled trials, single-arm studies, and retrospective analyses, to provide a comprehensive assessment of ADCs in treating urological cancers.

It seeks to offer additional therapeutic insights into the use of ADCs for treating diverse urological cancers and expand clinical treatment options. This article is presented in compliance with the PRISMA reporting checklist.

## 2 Methods

### 2.1 Search strategy

Literature on antibody-drug conjugates (ADCs) for treating advanced urological cancers was retrieved from seven databases: Embase, PubMed, Web of Science, Cochrane Library, CNKI, VIP database and WanFang data, with the final search conducted on 18 October 2024. The European Society of Medical Oncology’s (ESMO) meeting abstracts were also examined. The subject terms and free words looked up were “Urologic Neoplasms” OR “Carcinoma, Transitional Cell” OR “Kidney Neoplasms” OR “Carcinoma, Renal Cell” OR “Urinary Bladder Neoplasms” OR “Prostatic Neoplasms” OR “Testicular Neoplasms” AND “Immunoconjugates” OR “enfortumab vedotin” OR “sacituzumab govitecan” OR “disitamab vedotin” OR “Brentuximab Vedotin”. In addition, the original literature’s references were personally reviewed to make sure all pertinent papers were included. The Centre for Reviews and Dissemination (CRD) website (registration number CRD42024617523) makes the study protocol publicly accessible. We also searched for unpublished studies by contacting experts in the field and checking clinical trial registries (ClinicalTrials.gov, WHO International Clinical Trials Registry Platform, EU Clinical Trials Register) for ongoing or completed but unpublished trials. Authors of identified studies were contacted to obtain any additional unpublished data.

### 2.2 Selection criteria

Studies that satisfied the following inclusion requirements were added to the meta-analysis: 1) participants received urological cancer diagnoses that were either locally advanced or metastatic (urothelial, kidney, testicular or prostate) based on the ESMO guideline criteria for urological cancers (Powles et al., 2022; Powles et al., 2024b; Oldenburg et al., 2022; Parker et al., 2020); 2) treatments employing antibody-drug conjugates (ADCs) or combine immunotherapy with ADCs; 3) study types encompassed randomized controlled trials, non-randomized controlled trials, and retrospective studies; 4) outcomes reported patient-related metrics such as overall response rate (ORR), disease control rate (DCR), overall survival (OS), progression-free survival (PFS), duration of response (DOR), and adverse events (AEs). The Response Evaluation Criteria in Solid Tumors (RECIST) version 1.1 (Eisenhauer et al., 2009) was used to measure tumor remission. We excluded meta-analyses, reviews, guidelines, letters, consensus documents, editorials, conference abstracts, case reports, and animal studies. Two researchers (TL and XX) independently screened the articles using the predetermined criteria for inclusion and exclusion. The two reviewers discussed and resolved any discrepancies that arose during the screening process; two more investigators (YR and ZL) decided on any issues that remained.

### 2.3 Data extraction and quality assessment

Data were independently retrieved from all included studies by two researchers (TL and XX), who then assessed the methodological quality of each study. The extracted data encompassed the author’s name, year of publication, National Clinical Trials identifier, country, sample size, study type, cancer type, HER2 expression, median age, intervention, molecular target, payload, median follow-up time, prior therapy, metastasis or not, lines of previous therapy, and endpoints reported. ORR, DCR, DOR, PFS, OS, the frequency of any adverse events (AEs), and the occurrence of grade 3 or higher AEs were used to evaluate clinical efficacy and safety results. Depending on the format of the included studies, the quality assessment can be divided into three categories: The updated Cochrane Collaboration’s Risk of Bias tool (RoB 2.0) (Sterne et al., 2019) was used to assess the methodological quality and risk of bias of randomized controlled trials (RCTs), the methodological index for non-randomized studies (MINORS) (Slim et al., 2003) was used to evaluate single-arm experiments, and the Newcastle Ottawa Scale (NOS) (Stang, 2010) methodological index was used to evaluate cohort studies.

### 2.4 Statistical analysis

R version 4.4.0 was used to analyze the data for the meta-analysis. Heterogeneity was evaluated through the chi-squared test and the I^2^ statistic, with a p-value of <0.05 considered indicative of statistical significance. When there was significant heterogeneity (p < 0.05 and I^2^ > 50%), we used a random-effects model; otherwise, we employed a fixed-effects model. Sensitivity analyses were performed to evaluate the overall results’ stability and dependability. A funnel plot and Egger’s test were also used to investigate possible publication bias; a p-value of less than 0.05 indicates significance in statistics.

## 3 Results

### 3.1 Study selection

**FIGURE 1:**
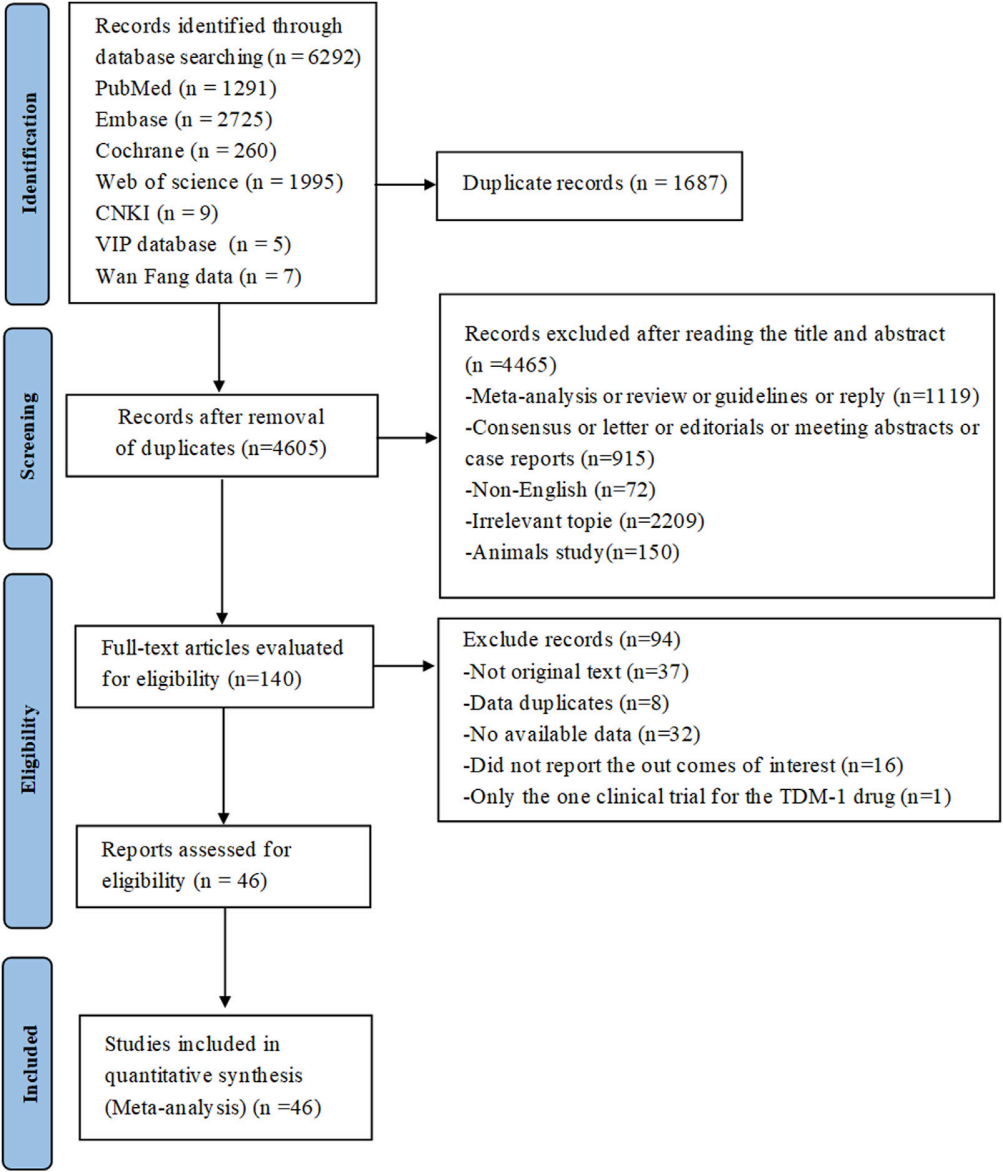
PRISMA flow diagram of the study process. PRISMA, Preferred Reporting Items for Systematic Reviews and Meta-Analysis.

The preliminary search across seven databases (PubMed = 1,291, Embase = 2,725, Cochrane Library = 260, Web of Science = 1,995, CNKI = 9, VIP database = 5 and WanFang data = 9) yielded a total of 6,292 published studies. 140 studies were kept for additional review after duplicates were eliminated and titles and abstracts were screened. Following full-text assessment, 94 studies were excluded for reasons such as the absence of original data or accessible information, data duplication, failure to report relevant outcomes, or inclusion of only a single clinical trial on the TDM-1 drug. In the end, the meta-analysis had 46 studies with 3,250 patients that satisfied the inclusion criteria (Kollmannsberger et al., 2021; Koshkin et al., 2022; Milowsky et al., 2016; Powles et al., 2024a; O'Donnell et al., 2023; Yu et al., 2021; Powles et al., 2021; Pet et al., 2024; Bardia et al., 2021; Xu et al., 2022; Xu et al., 2023; Endo et al., 2024; Minato et al., 2024; Rosenberg et al., 2019; Takahashi et al., 2020; Rosenberg et al., 2020; Siming Li et al., 2023; Zschäbitz et al., 2023; Miyake et al., 2024; Fukuokaya et al., 2024; Minato et al., 2023; Tagawa et al., 2021; Grivas et al., 2024; McGregor et al., 2024; Wei et al., 2023; Sheng et al., 2024; Xinan Sheng et al., 2023; Chen et al., 2023; Wasilijiang Wahafu et al., 2024; Zhu et al., 2024; Chen et al., 2024; Thompson et al., 2018; McGregor et al., 2020; Massard et al., 2019; Pal et al., 2019; Andrea Necchi et al., 2016; Ashkar et al., 2021; McHugh et al., 2019; Galsky et al., 2008; de Bono et al., 2021; Petrylak et al., 2019; Petrylak et al., 2020; Schatz et al., 2024; Shen et al., 2023; Danila et al., 2019; Aggarwal et al., 2022). Among these studies, 29 studies involved urothelial cancer, 5 studies related to renal cell carcinoma, 2 studies involved testicular cancer, 10 studies were on metastatic castration-resistant prostate cancer. The National Clinical Trials Registry made the complete study results available, so even if NCT02020135^67^ was not original, it was nonetheless included in the analysis. Figure 1 shows the flowchart that depicts the literature selection procedure. Of the patients included, 198 had renal cell carcinoma, 42 had testicular cancer, 478 had metastatic castration-resistant prostate cancer, and 2,532 had confirmed urothelial carcinoma. Table 1 and Supplementary Table S1 provide detailed information about each included study.

**TABLE 1:** Characteristics of the studies included in the meta-analysis.

Study	Year	Nation	Sample size		Study type	Cancer type	HER2 expression	Median age, years	Intervention	Endpoints
			Male	Female						
Endo et al. (2024)	2024	Japan	14	6	Retrospective Study (cohort)	Urothelial Cancer	NR	73.0 (61–85)	1.25 mg/kg EV	ORR, DCR, PFS, OS, AEs
Koshkin et al. (2022)	2022	America	205	55	Retrospective Study (cohort)	Urothelial Cancer	NR	71	EV	ORR, DCR, PFS, OS
Minato et al. (2024)	2024	Japan	61	19	Retrospective Study (cohort)	Urothelial Cancer	NR	73.0 (67–76)	1.25 mg/kg EV	ORR,DCR,PFS,OS,AEs
Yu et al. (2021)	2021	America	66	23	Singlearm Study	Urothelial Cancer	NR	75.0 (68–78)	1.25 mg/kg EV	ORR,DCR,DOR,PFS,OS,AEs
Rosenberg et al. (2019)	2019	America	88	37	Singlearm Study	Urothelial Cancer	NR	69.0 (40–84)	1.25 mg/kg EV	ORR,DCR,TOR,DOR,PFS,OS,AEs
O'Donnell et al. (2023)	2023	America	56	17	Randomized Study	Urothelial Cancer	NR	74.0 (56–89)	1.25 mg/kg EV	ORR,DCR,DOR,TOR,PFS,OS, AEs
									1.25 mg/kg EV + pembrolizumab	
Powles et al. (2021)	2021	America	238	63	Randomized Study	Urothelial Cancer	NR	68.0 (34–85)	1.25 mg/kg EV	ORR,TOR,DCR,DOR,PFS,OS,AEs
Takahashi et al. (2020)	2019	Japan	15	2	Singlearm Study	Urothelial Cancer	NR	67.0 (57,82)	1.00 mg/kg EV	ORR,DCR,AEs
									1.25 mg/kg EV	
Takahashi et al. (2020)	2020	America	111	44	Singlearm Study	Urothelial Cancer	NR	67.0 (24–86)	0.5,0.75,1.0,1.25 mg/kg	ORR,DCR,DOR,PFS,OS,AEs
Powles et al. (2024a)	2024	America	344	98	Randomized Study	Urothelial Cancer	NR	69.0 (37–87)	1.25 mg/kg EV + pembrolizumab	ORR,DCR,DOR,TOR,PFS,OS,AEs
Rosenberg et al. (2020)	2023	China	40		Singlearm Study	Urothelial Cancer	NR	NR	1.25 mg/kg EV	ORR,DCR,DOR,PFS,OS,AEs
Siming Li et al. (2023)	2023	Germany	87		Retrospective Study (cohort)	Urothelial Cancer	NR	66.0 (31–89)	1.25 mg/kg EV	ORR,DCR,PFS,OS,AEs
Zschäbitz et al. (2023)	2024	Japan	25	9	Retrospective Study (cohort)	Urothelial Cancer	NR	76.0 (64–86)	1.25 mg/kg EV	ORR,DCR,PFS,OS,AEs
Miyake et al. (2024)	2024	Japan	69	34	Retrospective Study (cohort)	Urothelial Cancer	NR	74.0 (69–79)	1.25 mg/kg EV	ORR,DCR,PFS,OS,AEs
Fukuokaya et al. (2024)	2023	Japan	22	4	Retrospective Study (cohort)	Urothelial Cancer	NR	73 (65–76)	1.25 mg/kg EV	ORR,DCR,PFS,OS,AEs
Minato et al. (2023)	2021	America	88	25	Singlearm Study	Urothelial Cancer	NR	66.0 (33–90)	10 mg/kg SG	ORR,DCR,DOR,TOR,PFS,OS,AEs
Tagawa et al. (2021)	2024	America	34	7	Singlearm Study	Urothelial Cancer	NR	67.0 (46–86)	10 mg/kg SG+ 200 mg pembrolizumab	ORR,DCR,DOR,TOR,PFS,OS,AEs
Pet et al. (2024)	2024	America	23	15	Singlearm Study	Urothelial Cancer	NR	73.0 (41–87)	10 mg/kg SG	ORR,DCR,DOR,TOR,PFS,OS,AEs
Grivas et al. (2024)	2024	America	18	5	Singlearm Study	Urothelial Cancer	NR	70.0 (63–76)	SG + EV	ORR,DCR,PFS,OS,AEs
Bardia et al. (2021)	2021	America	45		Singlearm Study	Urothelial Cancer	NR	NR	10 mg/kg SG	ORR,DCR,DOR,PFS,OS,AEs
McGregor et al. (2024)	2023	China	8	1	Retrospective Study (cohort)	Urothelial Cancer	Positive Negative	71.9 (60–84)	2 mg/kgRC-48 + 200 mg tislelizumab/3 mg/kg toripalimab	ORR,PFS,AEs
Wei et al. (2023)	2024	China	33	10	Singlearm Study	Urothelial Cancer	Positive	64.0 (45–75)	2.0 mg/kg DV (RC48-C005)	ORR,DCR,DOR,PFS,OS,AEs
		China	47	17	Singlearm Study		Positive	62.5 (40–79)	2.0 mg/kg DV (RC48-C009)	ORR,DCR,DOR,PFS,OS,AEs
Xu et al. (2022)	2022	China	19		Singlearm Study	Urothelial Cancer	Negative	64.0 (36–77)	2.0 mg/kgDV	ORR,DCR,PFS,AEs
Sheng et al. (2024)	2023	China	22	19	Singlearm Study	Urothelial Cancer	Positive Negative	66.0 (42–76)	2.0 mg/kgDV+3 mg/kg toripalimab	ORR,DCR,PFS,AEs
Xu et al. (2023)	2023	China	38		Retrospective Study (cohort)	Urothelial Cancer	Positive Negative	70.0 (38–93)	2.0 mg/kgDV+3 mg/kg toripalimab/+200 mg tislelizumab	ORR,DCR,DOR,PFS,AEs
								65.0 (49–77)	2.0 mg/kgDV	
Xinan Sheng et al. (2023)	2023	China	26	10	Retrospective Study (cohort)	Urothelial Cancer	Positive Negative	62.4 (47–87)	2.0 mg/kgDV + Immunotherapy	ORR,DCR,PFS,AEs
Chen et al. (2023)	2024	China	8	5	Singlearm Study	Urothelial Cancer	Positive Negative	72.0 (61–83)	2.0 mg/kgDV+ 6 mg/kg cadonilimab	ORR,DCR,AEs
Wasilijiang Wahafu et al. (2024)	2024	China	8	8	Retrospective Study (cohort)	Urothelial Cancer	Positive Negative	66 (51–81)	2.0 mg/kgDV+200 mg tislelizumab	ORR,DCR,AEs
Zhu et al. (2024)	2024	China	70	33	Retrospective Study (cohort)	Urothelial Cancer	Positive Negative	68 (35–93)	2.0 mg/kg DV	ORR,DCR,PFS,AEs
									2.0 mg/kg DV + immunotherapy	
									2.0 mg/kg DV + chemotherapy	
Chen et al. (2024)	2018	America	19	7	Singlearm Study	Renal Cell Carcinoma	NR	NR	AGS-16M8F(Hyb)	AEs
		America	27	7	Singlearm Study	Renal Cell Carcinoma				
Thompson et al. (2018)	2020	America	15	1	Singlearm Study	Renal Cell Carcinoma	NR	67.0 (56–84)	CDX-014	ORR,DCR,PFS,OS,AEs
Kollmannsberger et al. (2021)	2021	Canada	49	18	Randomized Study	Renal Cell Carcinoma	NR	63.0 (33–77)	1.8 mg/kg AGS-16C3F	ORR,DOR,PFS,DCR,OS,AEs
McGregor et al. (2020)	2019	France	32	5	Singlearm Study	Renal Cell Carcinoma	NR	NR	AMG 172	ORR,DCR,AEs
Massard et al. (2019)	2019	America	18	0	Singlearm Study	Renal Cell Carcinoma	NR	64.0 (47–74)	15,30,50 mg/kg SGN-CD70A	ORR,DCR,PFS,AEs
Pal et al. (2019)	2016	Italy	24		Singlearm Study	Testicular cancer	NR	NR	Brentuximab vedotin	ORR,AEs
Andrea Necchi et al. (2016)	2021	America	18		Singlearm Study	Testicular cancer	NR	34.7 (23.3–56.6)	Brentuximab vedotin	ORR,AEs
Ashkar et al. (2021)	2019	America	46	0	Singlearm Study	Metastatic castration-resistant prostate cancer	NR	69.5 (53,87)	ASG-5ME	ORR,DCR,AEs
Milowsky et al. (2016)	2016	America	62	0	Singlearm Study	Metastatic castration-resistant prostate cancer	NR	69.0 (52–84)	MLN2704	AEs
McHugh et al. (2019)	2008	America	23	0	Singlearm Study	Metastatic castration-resistant prostate cancer	NR	66.0 (53–81)	MLN2704	AEs
Galsky et al. (2008)	2021	UK	33	0	Singlearm Study	Metastatic castration-resistant prostate cancer	NR	71.0 (54–81)	MEDI3726	ORR,DCR,TOR,PFS,OS,DOR,AEs
de Bono et al. (2021)	2019	America	52	0	Singlearm Study	Metastatic castration-resistant prostate cancer	NR	70 (50–87)	PSMA ADC	AEs
Petrylak et al. (2019)	2020	America	119	0	Singlearm Study	Metastatic castration-resistant prostate cancer	NR	71 (50–91)	PSMA ADC	AEs
Results Posted on ClinicalTrials.gov (Petrylak et al., 2020)	NR	America	9	0	Singlearm Study	Metastatic castration-resistant prostate cancer	NR	74.6 (66–84)	PSMA ADC	ORR,DCR,AEs
Schatz et al. (2024)	2023	America	24	0	Singlearm Study	Metastatic castration-resistant prostate cancer	NR	NR	ARX517	AEs
Shen et al. (2023)	2019	America	77	0	Singlearm Study	Metastatic castration-resistant prostate cancer	NR	68 (43–88)	DSTP3086S	AEs
Danila et al. (2019)	2022	America	33	0	Singlearm Study	Metastatic castration-resistant prostate cancer	NR	NR	FOR46	AEs

### 3.2 Quality assessment

The updated Cochrane Collaboration’s Risk of Bias tool (RoB 2.0) (Sterne et al., 2019) was used to examine four randomized controlled trials (RCTs). Random sequence creation, allocation concealment, participant and staff blinding, outcome assessor blinding, insufficient outcome data, selective reporting, and other possible sources of bias are among the seven domains that are assessed by the tool. Each domain is rated as low risk, high risk, or some concerns. For random sequence generation, all RCTs used appropriate methods to generate random sequences, such as computer-generated random numbers, ensuring low risk of bias in this domain. Allocation concealment was adequately addressed in all studies, with methods like sealed envelopes or centralized randomization systems, maintaining low risk. Blinding of participants and personnel was not always possible due to the nature of the interventions, leading to some concerns in this domain for two studies. However, blinding of outcome assessment was successfully implemented in all RCTs, resulting in low risk. Incomplete outcome data was minimal, with no significant dropouts or missing data, thus maintaining low risk. Selective reporting was assessed by comparing the study protocol and the published results, and all studies were found to have low risk in this domain. Other potential biases, such as funding sources and conflicts of interest, were also evaluated and found to be low risk. All randomized controlled trials (RCTs) were judged to have low risk of bias. Twelve cohort studies were assessed using the Newcastle-Ottawa Scale (NOS) (Stang, 2010), with the evaluation concentrating on three main areas: selection of the study population (scored 0–4), comparability between groups (scored 0–2), and outcome measurement (scored 0–3). And the maximum total score is 9. All cohort studies scored well on selection and outcome measurement, with scores ranging from 7 to 9. The comparability between groups was adequately addressed in all studies, ensuring high quality. Detailed scores for each study are provided in Tables 2–4. Studies that achieved a total score of 6 or higher were considered to be of high quality, and all of the cohort studies met this criterion. The Methodological Index for Non-Randomized Studies (MINORS), which consists of 12 criteria for evaluation, was used to evaluate thirty single-arm studies. Eight of these criteria are specifically applicable to non-randomized controlled studies, including clearly defined study objectives, uniformity in patient inclusion, and anticipated data collection, among other aspects^34^. Each criterion is scored from 0 to 2, with a maximum score of 16. All single-arm studies scored well on clearly stated aims and appropriate endpoints. However, only 15 studies calculated the sample size, and 10 studies had a loss to follow-up rate of less than 10%. The overall quality scores ranged from 12 to 16, indicating high methodological quality.

**TABLE 2:** Quality assessment of the randomized studies included in the meta-analysis.

ROB2	Year	Q1	Q2	Q3	Q4	Q5	Total
12 Peter H. O’Donnell	2023	Low concerns	Low concerns	Low concerns	Low concerns	Low concerns	Low concerns
14 Thomas Powles	2021	Low concerns	Low concerns	Low concerns	Low concerns	Low concerns	Low concerns
50 Thomas Powles	2024	Low concerns	Low concerns	Low concerns	Low concerns	Low concerns	Low concerns
3 Christian Kollmannsberger	2021	Low concerns	Low concerns	Low concerns	Low concerns	Low concerns	Low concerns

Notes: Q1, bias due to randomization process; Q2, bias due to deviations from intended interventions; Q3, bias due to missing outcome data; Q4, bias in measurement of the outcome; Q5, bias due to selective reporting.

**TABLE 3:** Quality assessment of the single-arm studies included in the meta-analysis.

MINORS	Year	Q1	Q2	Q3	Q4	Q5	Q6	Q7	Q8	Total
1 Elisabeth G. E. de Vries	2023	2	2	1	2	2	2	2	2	15
11 Evan Y Yu	2021	2	2	2	2	2	2	1	2	15
13 Jonathan E. Rosenberg	2019	2	2	1	2	2	2	1	2	14
15 Shunji Takahashi	2019	2	2	1	2	2	1	1	2	13
16 Jonathan E. Rosenberg	2020	2	2	2	2	2	1	1	2	14
22 Siming Li	2023	2	2	1	2	2	1	1	2	13
32 Scott T. Tagawa	2021	2	2	2	2	2	2	1	2	15
34 Petros Grivas	2024	2	2	1	2	2	2	2	2	15
35 Daniel P. Petrylak	2024	2	1	2	2	2	2	1	2	14
33 B. A. McGregor	2023	2	1	2	2	2	1	1	2	13
36 A. Bardia	2021	2	2	2	2	2	2	2	2	16
30 John A. Thompson	2018	2	1	2	2	2	1	1	2	13
30 John A. Thompson	2018	2	2	2	2	2	1	1	2	14
31Bradley A. McGregor	2020	2	2	2	2	2	2	2	2	16
5 Christophe Massard	2019	2	2	2	2	2	1	1	2	14
10 Sumanta K. Pal	2019	2	1	2	2	2	1	2	2	14
19 Xinan Sheng	2023	2	2	2	2	2	1	1	2	14
19 Xinan Sheng	2023	2	2	2	2	2	1	2	2	15
20 Huayan Xu	2022	2	1	1	2	2	1	1	2	12
21 Xinan Sheng	2023	2	2	2	2	2	1	1	2	14
28 Wasilijiang	2024	2	2	1	2	2	1	1	2	13
45 Andrea Necchi	2016	2	1	2	2	2	0	1	2	12
46 Ryan Ashkar	2021	2	2	2	2	2	1	1	2	14
6 Deaglan McHugh	2019	2	2	2	2	2	1	1	2	14
7 Matthew I. Milowsky	2016	2	2	1	2	2	1	1	2	13
37 Matthew D. Galsky	2008	2	2	2	2	2	0	1	2	12
38 Johann S. De Bono	2021	2	2	2	2	2	2	2	2	16
39 Daniel P. Petrylak	2019	2	2	1	2	2	1	1	2	13
40 Daniel P. Petrylak	2019	2	2	2	2	2	1	1	2	14
41 Results Posted onClinicalTrials.gov	NR	2	2	2	2	2	1	1	2	14
42 J. Shen	2023	2	1	1	2	2	1	1	2	12
43 Daniel C. Danila	2019	2	1	2	2	2	1	1	2	13
44 Rahul Raj Aggarwal	2022	2	1	1	2	2	1	1	2	12

Notes: Q1, a stated aim of the study; Q2, inclusion of consecutive patients; Q3, prospective collection of data; Q4, end point appropriate to the study aim; Q5, unbiased evaluation of end points; Q6, follow-up period appropriate to the major end point; Q7, loss to follow-up not exceeding 5%; Q8, prospective calculation of the sample size.

**TABLE 4:** Quality assessment of the cohort studies included in the meta-analysis.

NOS	Year	Q1	Q2	Q3	Q4	Q5	Q6	Q7	Q8	Total
2 Yuki Endo	2024	1	1	1	1	1	1	1	1	8
4 Vadim S. Koshkin	2022	1	1	1	1	1	1	1	1	8
9 AKINORI MINATO	2024	1	1	1	1	1	1	1	1	8
23Stefanie Zscha¨bitz	2023	1	1	1	1	1	1	0	0	6
24 Makito Miyake	2024	1	1	1	1	1	1	0	1	7
25 Wataru Fukuokaya	2024	1	1	1	1	1	1	1	1	8
26 Jingwei Xu	2024	1	1	1	1	2	1	1	1	9
27 Meiting Chen	2023	1	1	1	1	1	1	0	1	7
29 Kejia Zhu	2024	1	1	1	1	1	1	1	1	7
48 Jinchao Chen	2024	1	1	1	1	2	1	0	1	8
49 AKINORI MINATO	2023	1	1	1	1	1	1	1	1	7
17 Yongbao Wei	2023	1	1	1	1	1	1	1	1	8

Notes: Q1, representativeness of the exposed cohort; Q2, selection of the non-exposed cohort; Q3, ascertainment of exposure; Q4, demonstration that outcome of interest was not present at start of study; Q5, comparability of cohorts on the basis of the design or analysis; Q6, assessment of outcome; Q7, was follow-up long enough for outcomes to occur; Q8, adequacy of follow-up of cohorts.

All of the studies included were categorized as low-risk, and the quality evaluation details are included in Tables 2–4.

### 3.3 Tumor response

#### 3.3.1 Urothelial cancer

**FIGURE 2:**
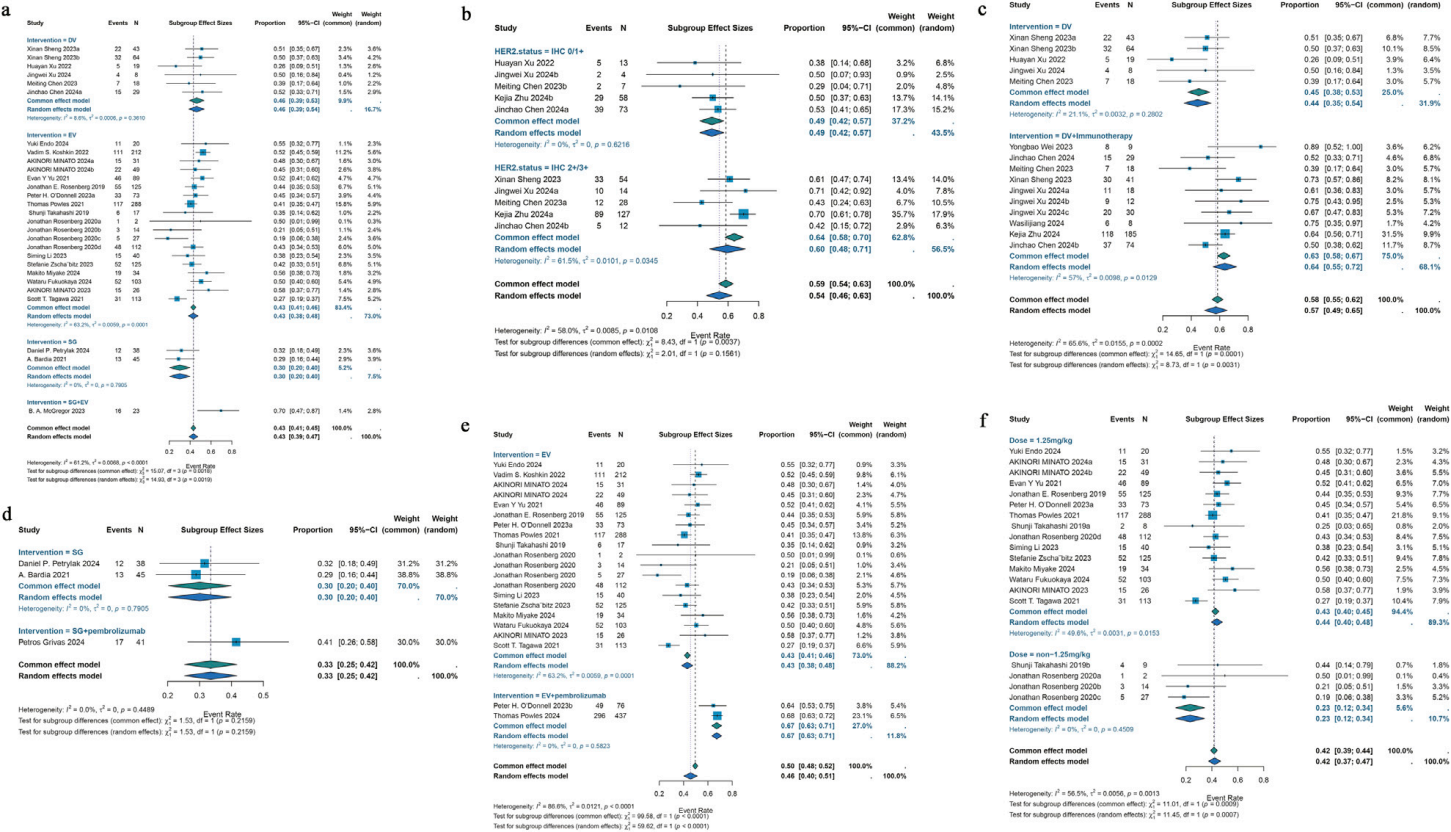
Forest plot for the pooled results of the different subgroup analysis in urothelial cancer studies. **(a)** ORR for intervention subgroup analysis (comparing EV, DV, SG, and combination therapies), **(b)** ORR for HER2 receptor status subgroup analysis (comparing IHC 0/1+ vs. IHC 2+/3+), **(c)** ORR for DV therapy whether combination with pembrolizumab therapy (comparing DV monotherapy vs. DV + pembrolizumab), **(d)** ORR for SG therapy whether combination with pembrolizumab therapy (comparing SG monotherapy vs. SG + pembrolizumab), **(e)** ORR for EV therapy whether combination with pembrolizumab therapy (comparing EV monotherapy vs. EV + pembrolizumab), **(f)** ORR for drug dose subgroup analysis (comparing 1.25 mg/kg vs. non-1.25 mg/kg EV dose). ORR, overall response rate; HER2, human epidermal growth factor receptor 2; DV, disitamab vedotin; SG, sacituzumab govitecan; EV, enfortumab vedotin.

**FIGURE 3:**
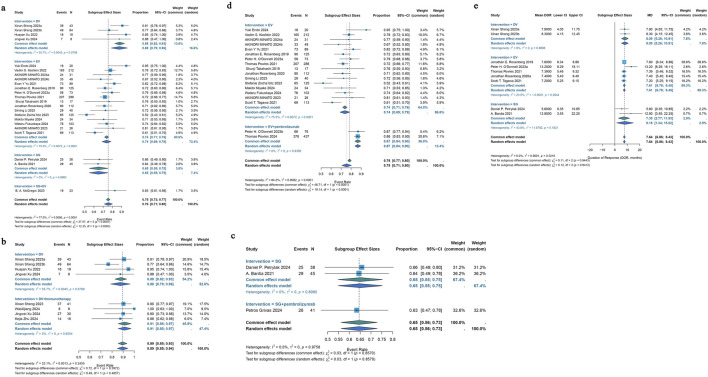
Forest plot for the pooled results of the different subgroup analysis in urothelial cancer studies. **(a)** DCR for intervention subgroup analysis, **(b)** DCR for DV therapy whether combination with pembrolizumab therapy, **(c)** DCR for SG therapy whether combination with pembrolizumab therapy, **(d)** DCR for EV therapy whether combination with pembrolizumab therapy, **(e)** DOR for intervention subgroup analysis in urothelial cancer studies. DCR, disease control rate; DOR, duration of response. DV, disitamab vedotin; SG, sacituzumab govitecan; EV, enfortumab vedotin.

23 studies that were part of the analysis assessed the efficacy of antibody-drug conjugates (ADCs) for urothelial cancer; the overall response rate (ORR) varied from 19% to 70%. Given the significant heterogeneity (I^2^ = 61.2%; p < 0.0001), a random-effects model was employed. The pooled ORR for all ADCs was 43% (95% CI: 39%–47%). Subgroup analyses were performed based on different interventions. The results revealed a pooled ORR of 46% (95% CI: 39%–53%) for patients treated with DV intervention, 43% (95% CI: 38%–48%) for those receiving EV intervention, 30% (95% CI: 20%–40%) for those treated with SG intervention, and 70% (95% CI: 47%–87%) for patients receiving a combination of SG and EV interventions (Figure 2a). Due to variations in HER2 status among urothelial cancer patients receiving DV treatment, a reanalysis of the studies was conducted. For the six studies reporting HER2 status, a subgroup analysis was performed based on IHC 0/1+ or IHC 2+/3+ classification. A pooled ORR of 49% (95% CI: 42%–57%) for patients with IHC 0/1+ and 60% (95% CI: 48%–71%) for patients with IHC 2+/3+ was found by the subgroup analysis (Figure 2b). Subgroup analysis was performed for the 29 studies that included various ADC monotherapies and combination therapies with immunotherapy. Patients treated with DV monotherapy had a pooled ORR of 45% (95% CI: 38%–53%), while those treated with DV in combination with immunotherapy had a pooled ORR of 64% (95% CI: 55%–72%) (Figure 2c). Patients receiving SG monotherapy had a pooled ORR of 30% (95% CI: 20%–40%), while those receiving SG combination therapy with pembrolizumab had a pooled ORR of 41% (95% CI: 26%–58%) (Figure 2d). Additionally, patients receiving EV monotherapy had a pooled ORR of 43% (95% CI: 38%–48%), while patients receiving EV combination therapy with pembrolizumab had a pooled ORR of 67% (95% CI: 63%–71%) (Figure 2e). For differences in drug dose on EV monotherapy, we also performed subgroup analysis. A pooled ORR of 43% (95% CI: 40%–45%) for individuals receiving 1.25 mg/kg and 23% (95% CI: 12%–34%) for patients taking a different dosage was found by subgroup analysis by doses of medication (Figure 2f). A pooled DCR of 76% (95% CI: 71%–80%) with considerable heterogeneity (I^2^ = 77.0%; p < 0.0001) was found after analyzing DCR data from 21 trials on ADCs. The subgroup analysis showed that patients receiving DV intervention had a pooled DCR of 88% (95% CI: 79%–96%), patients receiving EV intervention had a pooled DCR of 74% (95% CI: 69%–79%), patients receiving SG intervention had a pooled DCR of 65% (95% CI: 55%–75%), and patients receiving SG combination therapy with EV intervention had a pooled DCR of 83% (95% CI: 61%–95%) (Figure 3a). For the 25 studies of including different ADC drugs monotherapy and combination therapy with immunotherapy, subgroup analysis was performed. The subgroup analysis by ADCs drugs monotherapy or combination therapy indicated a pooled DCR of 88% (95% CI: 79%–96%) for DV monotherapy patients and 91% (95% CI: 85%–97%) for DV combination therapy with immunotherapy patients (Figure 3b). A pooled DCR of 65% (95% CI: 55%–75%) was observed for patients receiving SG monotherapy, and 63% (95% CI: 47%–78%) for those treated with SG combination therapy with pembrolizumab (Figure 3c). Similarly, the pooled DCR was 74% (95% CI: 69%–79%) for patients on EV monotherapy, and 87% (95% CI: 84%–90%) for patients receiving EV in combination with pembrolizumab (Figure 3d). Eight of the 46 studies that were part of the study revealed a median DOR (I^2^ = 0.0%; p = 0.5210). All ADCs had a pooled median DOR of 7.64 months (95% CI: 6.86–8.43). According to subgroup analysis, the DV group’s pooled median DOR was 8.09 months (95% CI: 5.26–10.91), the EV group’s was 7.61 months (95% CI: 6.78–8.45), and the SG group’s was 7.35 months (95% CI: 2.77–11.93) (Figure 3e).

#### 3.3.2 Renal cell carcinoma

**FIGURE 4:**
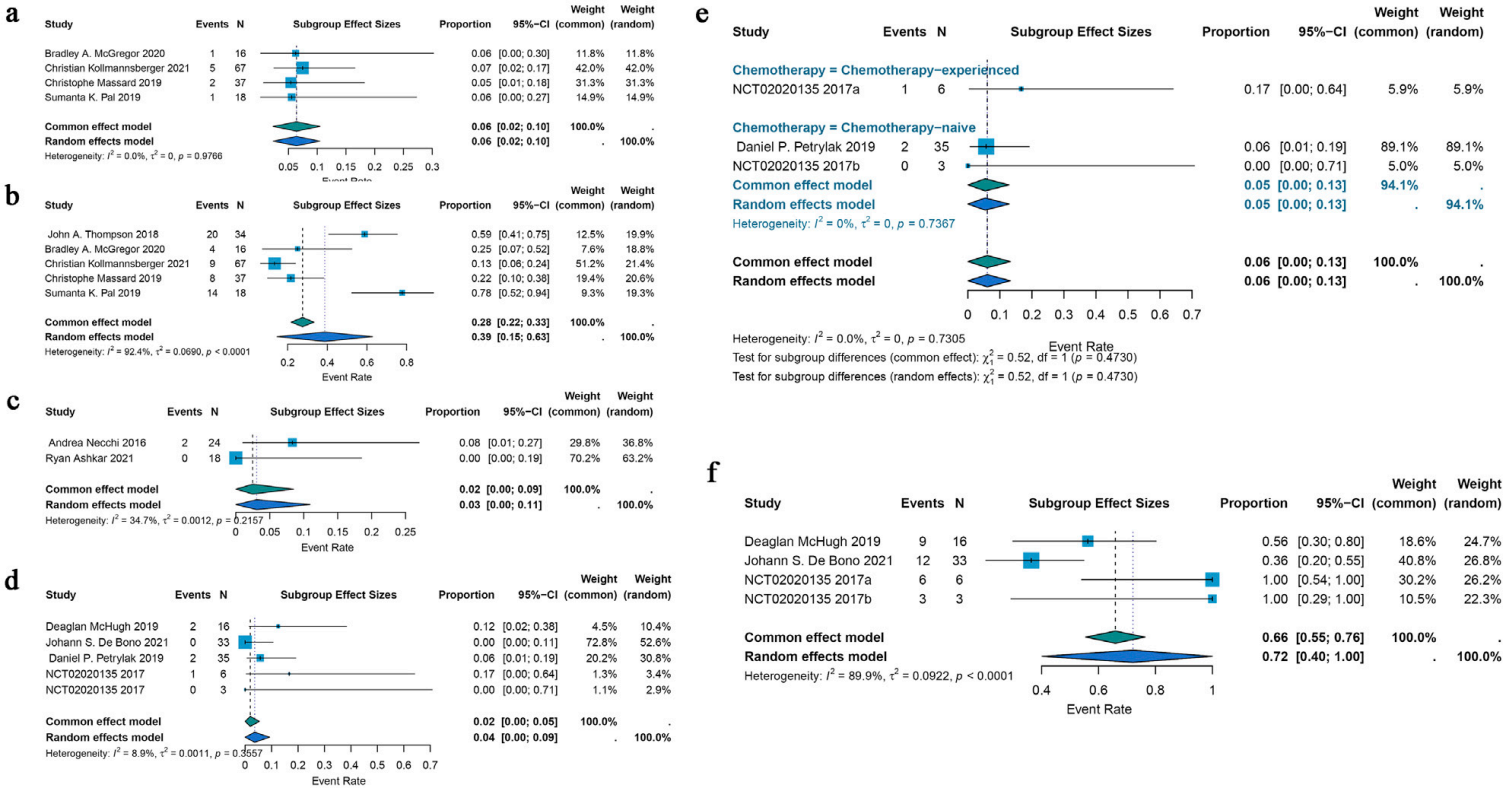
Forest plot for the pooled results of ORR, DCR in other urological cancers. **(a)** The pooled ORR for renal cell carcinoma studies, **(b)** the pooled DCR for renal cell carcinoma studies, **(c)** the pooled ORR in testicular cancer studies, **(d)** the pooled ORR in metastatic castration-resistant prostate cancer, **(e)** ORR in chemotherapy-experienced group and chemotherapy-naive group for metastatic castration-resistant prostate cancer, **(f)** the pooled DCR in metastatic castration-resistant prostate cancer. ORR, overall response rate; DCR, disease control rate.

Five included studies demonstrated that ADCs were effective in treating renal cell cancer, with ORRs ranging from 5% to 7%. The pooled ORR was 6% (95% CI: 2%–10%) after a common-effects model was utilized (I^2^ = 0.0%; p = 0.9766) (Figure 4a). A pooled DCR of 39% (95% CI: 15%–63%) was found by analyzing the DCR data from these 5 trials; there was also notable heterogeneity (I^2^ = 92.4%; p < 0.0001) (Figure 4b).

#### 3.3.3 Testicular cancer

Only two included studies evaluated the efficacy of ADCs for testicular cancer, and their ORRs varied from 0% to 8%. A common effects model was employed in the research (I^2^ = 34.7%; p = 0.2157). Using the identical brentuximab vedotin of ADCs, the study revealed a pooled ORR of 2% (95% CI: 0%–9%) (Figure 4c).

#### 3.3.4 Metastatic castration-resistant prostate cancer

The efficacy of ADCs for metastatic castration-resistant prostate cancer was assessed in four studies that were part of the analysis; the ORR spanned 0%–17%. The pooled ORR for all ADCs was 2% (95% CI: 0%–5%) using a common-effects model (I^2^ = 8.9%; p = 0.3557) (Figure 4d). A subgroup analysis based on the various forms of chemotherapy was conducted. According to this study, individuals with chemotherapy-experience had a pooled ORR of 17% (95% CI: 0%–64%) while patients without chemotherapy had a pooled ORR of 5% (95% CI: 0%–13%) (Figure 4e). A pooled DCR of 72% (95% CI: 40%–100%) with considerable heterogeneity (I^2^ = 89.9%; p < 0.0001) was seen when DCR data from three trials on ADCs were analyzed (Figure 4f).

### 3.4 Survival

#### 3.4.1 Urothelial cancer

**FIGURE 5:**
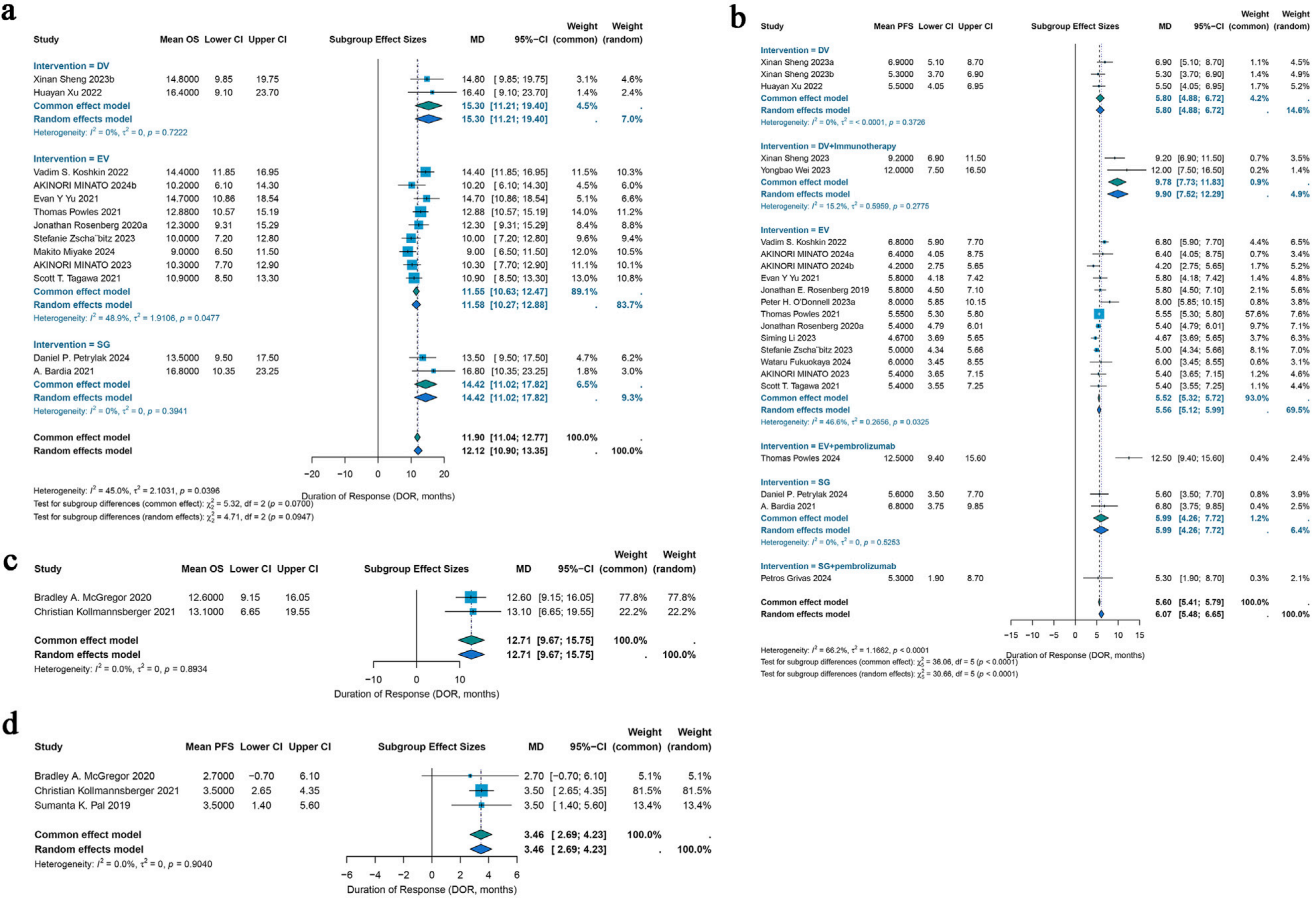
Forest plots for the pooled results of OS and PDS in urological cancer studies. **(a)** OS for different interventions subgroup analysis in urothelial cancer, **(b)** PFS for subgroup analysis of different monotherapy interventions and combination therapy with pembrolizumab interventions in urothelial cancer. **(c)** The pooled OS in renal cell carcinoma, **(d)** the pooled PFS in renal cell carcinoma. OS, overall survival; PFS, progress free survival.

For urothelial cancer, complete median OS data could only be extracted from 13 studies. Using a common-effects model, the pooled median OS was 11.90 months (95% CI: 11.04–12.77) (I^2^ = 45.0%; p = 0.0396). Analysis of subgroups was done based on interventions. Subgroup analysis showed a pooled median OS of 15.30 months (95% CI: 11.21–19.40) in patients with DV intervention, 11.55 months (95% CI: 10.63–12.47) in patients with EV intervention, and 14.42 months (95% CI: 11.02–17.82) in patients with SG intervention (Figure 5a). Of the 46 included trials, 20 studies had entire median PFS data that could be retrieved. In a random-effects model, the pooled median PFS was 6.07 months (95% CI: 5.48–6.65) (I^2^ = 66.2%; p < 0.0001). Subgroup analysis was conducted according to interventions. Subgroup analysis showed a pooled median PFS of 5.80 months (95% CI: 4.88–6.72) for DV monotherapy patients, 9.78 months (95% CI: 7.73–11.83) for DV combination therapy with immunotherapy patients, 5.52 months (95% CI: 5.32–5.72) for patients with EV monotherapy, 12.50 months (95% CI: 9.40–15.60) for patients with EV combination therapy with pembrolizumab, 5.99 months (95% CI: 4.26–7.72) for patients with SG monotherapy, 5.30 months (95% CI: 1.90–8.70) for patients with SG combination therapy with pembrolizumab (Figure 5b).

#### 3.4.2 Renal cell carcinoma

For renal cell carcinoma, median OS data were fully obtainable from only two studies, yielding a pooled median OS of 12.71 months under a common-effects model (95% CI: 9.67–15.75) with no observed heterogeneity (I^2^ = 0.0%; p = 0.8934) (Figure 5c). Among the 46 studies included, median PFS data were completely available in just three studies, resulting in a pooled median PFS of 3.46 months, also calculated using a common-effects model (95% CI: 2.69–4.23), with heterogeneity similarly absent (I^2^ = 0.0%; p = 0.9040) (Figure 5d).

### 3.5 Toxicity

The analysis focused on the most frequent and clinically significant adverse events (AEs), including both all-grade and grade ≥ III, associated with urothelial cancer treatments. These interventions comprised EV monotherapy, EV combined with pembrolizumab, DV monotherapy, and DV in combination with pembrolizumab (Table 5), most patients had grade 1 or 2 adverse effects while undergoing treatment. With rates of 58% (95% CI: 54%–62%), 34% (95% CI: 22%–47%), and 34% (95% CI: 24%–43%), respectively, alopecia, peripheral sensory neuropathy, and dysgeusia were the three most frequently reported adverse effects in the EV monotherapy group. For EV combination therapy with pembrolizumab, peripheral sensory neuropathy, rash, and fatigue, with incidences of 38% (95% CI: 29%–46%), 52% (95% CI: 27%–78%), and 45% (95% CI: 17%–73%) were the most common adverse events. In the DV monotherapy group, anemia, alopecia, and neutropenia were more frequently reported, with corresponding rates of 44% (95% CI: 14%–74%), 42% (95% CI: 34%–50%), and 42% (95% CI: 33%–51%). Peripheral sensory neuropathy, anemia, and decreased appetite were the most common adverse events (AEs) for DV combined therapy with pembrolizumab, occurring in 54% (95% CI: 25%–83%), 43% (95% CI: 6%–81%), and 38% (95% CI: 29%–46%).

**TABLE 5:** Pooled results of all adverse events of ADCs in urothelial cancer.

AEs	Intervention			
	1.25 mg/kg EV	1.25 mg/kg EV + Pembrolizumab	2.0 mg/kg DV	2.0 mg/kg DV + Pembrolizumab
Alopecia, % (95% CI)	38 (29–46)	39 (26–51)	42 (34–50)	36 (22–49)
Anemia, % (95% CI)	18 (11–24)	14 (11–17)	44 (14–74)	54 (25–83)
Decreased appetite, % (95% CI)	31 (18–45)	27 (23–31)	38 (29–46)	38 (29–46)
Diarrhea, % (95% CI)	23 (13–33)	28 (24–32)		
Dysgeusia, % (95% CI)	28 (19–38)	45 (17–73)		
Fatigue, % (95% CI)	34 (24–43)	42 (16–69)	41 (25–56)	26 (9–42)
Hyperglycemia, % (95% CI)	6 (4–9)	13 (10–16)		
Nausea, % (95% CI)	27 (15–40)	21 (17–24)	25 (18–31)	24 (5–42)
Neutropenia, % (95% CI)	13 (5–22)	9 (7–12)	42 (33–51)	
Peripheral sensory neuropathy, % (95% CI)	34 (22–47)	58 (54–62)		43 (6–81)
Pruritus, % (95% CI)	25 (12–37)	40 (36–44)		
Rash, % (95% CI)	24 (12–37)	52 (27–78)		
Vomiting, % (95% CI)			17 (11–23)	24 (7–42)

Notes: AE, adverse event; ES, effect size.

All ADCs were examined collectively to evaluate metastatic castration-resistant prostate cancer and renal cell carcinoma overall safety (Tables 6, 7). Fatigue 50% (95% CI: 36%–64%), thrombocytopenia 45% (95% CI: 30%–59%), and nausea 42% (95% CI: 32%–53%) were the most frequent adverse events (AEs) among patients with renal cell carcinoma who received ADCs. Furthermore, peripheral neuropathy was the most frequent adverse event (AE) among patients with metastatic castration-resistant prostate cancer undergoing ADCs (58%; 95% CI: 40%–76%), fatigue (44%; 95% CI: 29%–59%), and nausea (40%; 95% CI: 28%–53%).

**TABLE 6:** Pooled results of adverse events of ADCs in renal cell carcinoma.

AEs	Number of study	All grade			Number of study	≥ Grade III		
		ES, % (95CI)	I^2^%	p		ES, % (95CI)	I^2^%	p
Anemia	4	34 (20–49)	69.0	0.0217	3	13 (7–19)	0.0	0.3999
Constipation	3	27 (20–34)	0.0	0.8602				
Decreased appetite	4	34 (20–48)	73.7	0.0096				
Epistaxis	3	21 (14–28)	0.0	0.9938				
Fatigue	5	50 (36–64)	73.1	0.0023	2	9 (3–14)	0.0	0.5008
Headache	3	24 (17–31)	0.0	0.4259				
Nause	5	42 (32–53)	50.4	0.0731	2	2 (0–5)	4.1	0.3071
Peripheral edema	3	17 (11–24)	50.3	0.1339				
Pyrexia	3	26 (17–36)	0.0	0.6214				
Thrombocytopenia	3	45 (30–59)	62.0	0.0484	3	15 (9–22)	0.0	0.7814
Vomitting	4	32 (25–39)	43.9	0.1481	2	2 (0–5)	4.1	0.3071

Notes: AE, adverse event; ES, effect size.

**TABLE 7:** Pooled results of adverse events of ADCs in metastatic castration-resistant prostate cancer.

AEs	Number of study	All grade			Number of study	≥ Grade III		
		ES, % (95CI)	I^2^%	p		ES, % (95CI)	I^2^%	p
Anemia	4	4 (2–7)	44.5	0.1443				
Anorexia	3	32 (24–39)	11.6	0.3228				
Constipation	7	26 (16–37)	89.9	<0.0001	2	2 (0–4)	0.0	0.3514
Diarrhea	6	27 (22–32)	48.2	0.0858				
Dyspnea	4	19 (7–31)	86.8	<0.0001	3	2 (0–4)	0.0	0.8420
Fatigue	9	44 (29–59)	97.1	<0.0001	3	6 (2–9)	48.8	0.1419
Musculoskeletal pain	3	13 (7–18)	8.9	0.3336				
Nause	7	40 (28–53)	77.5	0.0002	2	2 (0–4)	0.0	0.7271
Peripheral neuropathy	4	58 (40–76)	78.9	0.0026	2	5 (1–8)	0.0	0.5036
Vomitting	5	22 (16–27)	0.0	0.6907				

Notes: AE, adverse event; ES, effect size.

The incidence of high-grade AEs (above grade III) was small (Tables 6–8). Among patients with urothelial cancer, the incidence of neutropenia was 12% (95% CI: 6%–19%) in 1.25 mg/kg EV group, which is the most frequent high-grade AEs. And the incidences of thrombocytopenia and anemia were 15% (95% CI: 9%–22%) and 13% (95% CI: 7%–19%) respectively for renal cell carcinoma patients, which are the highest incidence of high-grade AEs.

**TABLE 8:** Pooled results of adverse events ≥ grade III of ADCs in urothelial cancer.

AEs	Intervention	
	1.25 mg/kg EV	1.25 mg/kg EV + Pembrolizumab
Anemia, % (95% CI)	7 (4–10)	4 (2–5)
Decreased appetite, % (95% CI)	1 (0–2)	
Diarrhea, % (95% CI)	3 (1–5)	4 (2–6)
Fatigue, % (95% CI)	3 (2–4)	5 (0–11)
Hyperglycemia, % (95% CI)	3 (2–5)	6 (4–8)
Nausea, % (95% CI)	1 (0–2)	
Neutropenia, % (95% CI)	12 (6–19)	5 (3–7)
Peripheral sensory neuropathy, % (95% CI)	3 (1–5)	3 (0–6)
Pruritus, % (95% CI)	1 (0–3)	1 (0–2)
Rash, % (95% CI)	5 (1–8)	10 (0–28)

Notes: AE, adverse event; ES, effect size.

### 3.6 Sensitivity analysis

In order to evaluate the reliability of the pooled effect sizes for ORR, DCR, DOR, OS, and PFS in patients with urothelial cancer, ORR, DCR, and PFS in patients with renal cell carcinoma, ORR in patients with testicular cancer, and ORR and DCR in patients with metastatic castration-resistant prostate cancer, we performed sensitivity analyses for outcomes with I^2^ >50% by sequentially excluding individual studies.

The sensitivity analysis of all included studies showed that the pooled results with 95% CI for both the DV and EV intervention groups in urothelial cancer, as well as the metastatic castration-resistant prostate cancer group, were not notably influenced by any single study. This implies that the meta-analysis’s overall conclusions are solid and trustworthy. Supplementary Figure S1 displays the sensitivity analysis’s comprehensive findings.

### 3.7 Publication bias

In this study, publication bias was evaluated using funnel plots and Egger’s test. For the included urothelial cancer studies, the combined ORR (Egger’s test: p = 0.9173), DCR (Egger’s test: p = 0.828), median DOR (Egger’s test: p = 0.167) and median OS (Egger’s test: p = 0.0782) demonstrated no publication bias, but its median PFS (Egger’s test: p = 0.0469) had detected publication bias, but this did not affect the overall conclusions. For studies of renal cell carcinoma, the combined ORR (Egger’s test: p = 0.514), DCR (Egger’s test: p = 0.1399), and median PFS (Egger’s test: p = 0.4339) demonstrated no publication bias. About metastatic castration-resistant prostate cancer studies, the combined ORR (Egger’s test: p = 0.1455) and DCR (Egger’s test: p = 0.4876) demonstrated no publication bias.

Due to the limited number of included studies, it was not possible to assess the combined ORR of patients with testicular cancer and the median OS of patients with renal cell carcinoma for publication bias. Supplementary Figure S2 provides a full account of the publishing bias results. Most AEs were free of publication bias, but publication bias was present for decreased appetite, dysgeusia, hyperglycemia, nausea and neutropenia in EV monotherapy group of urothelial cancer studies, constipation in renal cell carcinoma studies and constipation, dyspnea, fatigue in metastatic castration-resistant prostate cancer studies, among all grades AEs. Among the grade ≥ III AEs, publication bias was observed for decreased appetite, diarrhea, fatigue, nausea, peripheral sensory neuropathy in EV monotherapy group of urothelial cancer studies.

## 4 Discussion

With over 100 ADCs currently in clinical research and 11 FDA-approved drugs currently in clinical utilization, ADCs are effective therapies for many kinds of cancers (Bordeau et al., 2023). They have the ability to minimize off-target effects while delivering strong cytotoxic chemicals straight to tumor cells. ADCs, first proposed by Ehrlich, enable selective binding tumor cells by fusing the effectiveness of cytotoxic medications with the accuracy of targeted antibodies. Because of the antibodies’ remarkable specificity and affinity for specific epitopes on the target antigen, this method enables the highly effective delivery of medicines, improving the index of therapy (Marei et al., 2022; Strebhardt and Ullrich, 2008; Hoffmann et al., 2018). Currently, three FDA-approved ADCs have been approved for the treatment of urological cancer. They are EV, SG, and RC-48. Compared with chemotherapy, pembrolizumab plus enfortumab vedotin showed a 55% lower risk of progressive disease or mortality. Furthermore, compared to the chemotherapy group, the percentage of urothelial carcinoma patients who showed an overall response was substantially higher in the enfortumab vedotin-pembrolizumab group (Powles et al., 2024a). In patients with urothelial cancer, single-agent EV produced a remarkable and encouraging outcome of 43% (Rosenberg et al., 2020). Reduced PSA and/or CTCs in individuals with metastatic castration-resistant prostate cancer were linked to prostate-specific membrane antigen (PSMA) (Petrylak et al., 2019). All of the aforementioned findings underscore the robust efficacy of ADC therapy in treating urothelial cancers. Nevertheless, some research findings contradict this conclusion. When comparing AGS-16C3F to axitinib, the PFS of patients with renal cell carcinoma of any histology was not improved. The findings demonstrated that, in comparison to axitinib, AGS-16C3F did not increase PFS (Kollmannsberger et al., 2021). Based on existing research, we have found that almost all patients experienced adverse reactions at any level. Compared to the usual therapy group, the ADC groups experienced fewer treatment-related adverse events of grade 3 or higher (Powles et al., 2024a). But some studies showed the incidence of some ≥ grade III adverse reactions, such as diarrhea, have the opposite effect (Kollmannsberger et al., 2021). Therefore, in light of the advancements in clinical trials, a meta-analysis, which is a high-quality evidence evaluation, reviewing the safety and efficacy of various ADC types in the treatment of advanced urological cancers is desperately needed. A total of 3,250 patients were included in this meta-analysis, which comprised 30 single-arm studies, 12 retrospective cohort studies, and 4 randomized controlled trials. We comprehensively assessed the differences in efficacy across various subgroups and examined the safety profile of ADCs in treating advanced urological cancers. Among the included studies, twenty-eight focused on advanced urothelial cancer, six on renal cell carcinoma, two on testicular cancer, and ten on metastatic castration-resistant prostate cancer. ADCs produced encouraging ORR and DCR in urothelial cancer, according to the pooled analysis, with PFS, OS, and DOR offering insightful information. All things considered, the results show that ADCs have a solid safety record and good therapeutic efficacy when used to treat these types of cancer.

For urothelial cancer, regardless of the interventions (including HER2 status, monotherapy, combination therapy with immunotherapy, and drug dosage), the pooled results from all studies showed an overall response rate (ORR) of 43% (95% CI: 39%–47%) and a disease control rate (DCR) of 76% (95% CI: 71%–80%). All treatment groups achieved median survival times, with a pooled median progression-free survival (PFS) of 6.07 months (95% CI: 5.48–6.65) and a median overall survival (OS) of 11.90 months (95% CI: 11.04–12.77). Additionally, the median duration of response (DOR) for urothelial cancer patients was 7.64 months. Subgroup analysis suggested higher ORR with SG-EV combination (70%) versus single-agent SG (30%) or EV (43%) and higher DCR (83% vs. 74%, 65%) than patients with DV intervention and EV intervention, indicating that SG combination with EV treatment was likely to have a better effect on advanced urothelial cancer. These results highlight the potential of ADCs as a first-line treatment option for patients with advanced urothelial cancer. Mutations in the HER2 gene are common in many types of cancer and play a major role in the development of new tumors and the spread of existing ones. Mechanistically, HER2 overexpression increases receptor clustering and subsequent endocytosis, promoting more efficient cellular uptake of DV (Majumdar and Siahaan, 2012). Furthermore, as demonstrated in HER2-overexpressing ovarian cancer models, accelerated internalization leads to faster lysosomal trafficking where proteolytic enzymes more efficiently cleave the linker, resulting in significantly higher intra-tumoral MMAE concentrations compared to serum levels (Zhu et al., 2021).Since overexpression of HER2 is connected to more aggressive disease, a higher risk of metastasis, and lower overall survival rates, it is strongly associated with a bad prognosis in urothelial cancer (Jimenez et al., 2001; Gan et al., 2021). Studies have indicated that patients receiving DV treatment with HER2 IHC 3+ expression tend to derive greater clinical benefits compared to those with HER2 IHC 2+ expression (Lei et al., 2023). This suggests that higher levels of HER2 overexpression may enhance the therapeutic efficacy of DV treatment in urothelial cancer patients. The powerful and remarkable efficiency of DV was also revealed by the ORR of 60% in IHC 2+/3+ expression compared to 49% in IHC 0/1+ expression of urothelial cancer. For the 29 studies of including different ADCs monotherapy and combination therapy with immunotherapy, subgroup analysis was performed. The US Food and Drug Administration (FDA) has approved EV monotherapy for the treatment of patients with locally advanced (LA) or metastatic urothelial cancer (mUC) who have had at least one course of therapy and are not eligible for cisplatin-based chemotherapy. The favorable results seen in cohort 2 of the phase II EV-201 research served as the foundation for its authorization (Yu et al., 2021; Pet et al., 2024). The combination of EV and pembrolizumab was approved as the initial treatment for individuals with mUC who are ineligible for cisplatin and whose condition worsens after taking a checkpoint inhibitor (CPI) (Food and Administration, 2023). In 2015, it was suggested that ADC and immunotherapy might work better in combination (Gerber et al., 2016). Subsequent clinical data have confirmed this view, with the combination of ADC and immunotherapy providing significant clinical benefit to patients in several clinical trials for different types of cancer, including breast, lung and urothelial cancers. Through processes like immunogenic cell death, antibody-dependent cell-mediated cytotoxicity, and dendritic cell activation, ADCs interact with immune cells and cancer. These interactions can work in concert with immunotherapy. ADCs specifically promote T cell infiltration into the tumor microenvironment by stimulating tumor-specific adaptive immunity. Immunocheckpoint inhibitors, meanwhile, aid in reviving worn-out T cells, boosting anticancer immune responses even more (Nicolò et al., 2022). We found that the DCR of almost all patients receiving combination therapy with immunotherapy were higher than those receiving monotherapy. Compared to ADCs monotherapy, the combination therapy even more nearly doubled the figures of median PFS and ORR in urothelial cancer patients. From the data we analyzed, the ORR, DCR, DOR and OS of the DV group were better than those of the EV and SG groups. A humanized IgG1 monoclonal antibody that targets Nectin-4 is combined with a microtubule-disrupting agent, monomethyl auristatin E (MMAE), and a cleavable mc-val-cit-PABC linker to form EV, an ADC. Another ADC is SG, which is made up of a topoisomerase I inhibitor (SN-38) coupled to an anti-Trop-2 antibody (sacituzumab) via the hydrolyzable CL2A linker. Disitamab vedotin (DV/RC48) is an ADC that uses a monoclonal antibody (hertuzumab) to target the human epidermal growth factor receptor 2 (HER2) and is connected to MMAE by a mc-val-cit-PABC linker (Yu et al., 2023). By preferentially delivering MMAE to HER2-positive cells or tumor tissues, RC48-ADC has strong targeted delivery and release capabilities. This leads to less off-target toxicity and increased anti-tumor activity, which in turn reduces systemic toxicity and improves therapeutic efficacy (Li et al., 2020). The efficacy of an antibody-drug conjugate (ADC) is significantly influenced by the specificity and affinity of the antibody for the tumor antigen, as well as the dynamics of protein turnover between the cell membrane and cytoplasm (Hamilton et al., 2022). These factors contribute to the efficient targeting and internalization of the ADC, which are essential for delivering the therapeutic payload to the cancer cells and ensuring effective treatment outcomes. For safety and efficacy considerations, IgG1 antibodies are commonly employed in the construction of the immunoglobulin component of antibody-drug conjugates (ADCs). This choice helps minimize hypersensitivity reactions and enhances immune-mediated cytotoxicity. Additionally, the stability of the payload is largely determined by the linker used, which plays a critical role in controlling the timing of drug release. An optimal linker ensures that the therapeutic payload is released at the target site, avoiding premature or delayed drug activation that could compromise treatment effectiveness (Emens et al., 2019; Hamilton et al., 2021; Emens et al., 2020). Otherwise, the quantity of cytotoxic molecules attached to each antibody, or the drug-to-antibody ratio that occurs affects the drug’s stability and therapeutic efficacy (Emens et al., 2019). Finally, the payload plays a crucial role in inducing the direct cytotoxic effect (Fu et al., 2022; Peters et al., 2024). For differences in drug dose on EV monotherapy, we also performed subgroup analysis. EV should be administered intravenously on days 1, 8, and 15 of a cycle (28 days) at a dose of 1.25 mg/kg (up to a dose 125 mg) until the disease progresses or the toxicity becomes intolerable for both monotherapy and combination therapy. We also investigated whether varying doses of EV would lead to differences in efficacy. The results of our subgroup analysis revealed a higher overall response rate (ORR) in the 1.25 mg/kg dose group (43%, 95% CI: 40%–45%) compared to the non-1.25 mg/kg dose group (23%, 95% CI: 12%–34%). These findings suggest that a dose of 1.25 mg/kg of EV is more effective and may be preferable for urothelial cancer patients. ADCs had a hopeful DCR for renal cell carcinoma, according to the pooled analysis, while PFS and OS also had a reference value that demonstrated good efficacy and consistent safety. An ORR and DCR of 6% (95% CI: 2%–10%) and 39% (95% CI: 15%–63%), respectively, were included in the combined findings of all five investigations. Only three studies showed a median OS of 12.71 (95% CI: 9.67–15.75) months and two studies showed a median PFS of 3.46 (95% CI: 2.69–4.23) months. This suggests that further research is needed to identify more effective ADCs or combination therapies for this cancer type. Similarly, the limited number of studies on testicular cancer and metastatic castration-resistant prostate cancer highlights the need for additional clinical trials to fully assess the potential of ADCs in these settings. For testicular cancer, only two studies treated with brentuximab vedotin were included showing an ORR of 2% (95% CI: 0%–9%). Therefore, we conclude that ADCs may have an important role in the treatment of renal cell carcinoma and testicular cancer, but more data are needed to support it. With regard to metastatic castration-resistant prostate cancer, though there are no ADCs approved for prostate cancer, there are some ADCs targeting different antigens in clinical studies, such as PSMA, TROP-2, STEAP1, TF and DLL-3 (Sardinha et al., 2023). In our meta-analysis, the pooled results from all four studies revealed an ORR of 2% (95% CI: 0%–5%) and a DCR of 72% (95% CI: 40%–100%). We performed subgroup analyses to look more closely at how previous chemotherapy affected study results. The pooled ORR for chemotherapy-experienced patients was 17% (95% CI: 0%–64%), whereas the ORR for chemotherapy-naïve patients was 5% (95% CI: 0%–13%). Thus, chemotherapy-experienced patients demonstrated a higher overall response rate (ORR) compared to chemotherapy-naïve patients. Taken together, antibody-drug conjugates (ADCs) offer patients with advanced urological cancers new therapeutic alternatives with regard to clinical efficacy. According to our research of adverse events (AEs), almost all patients had at least one occurrence; in cases with urothelial cancer, peripheral sensory neuropathy, alopecia, and anemia were the most frequent. Most of the adverse reactions that occurred ≥ grade III had an incidence of less than 10%, which was greatly reduced. This indicates that ADCs are generally well-tolerated, although careful monitoring and management of side effects are essential. The most common AEs for renal cell carcinoma patients receiving ADCs were fatigue 50% (95% CI: 36%–64%), thrombocytopenia 45% (95% CI: 30%–59%) and nausea 42% (95% CI: 32%–53%). Additionally, peripheral neuropathy was the most frequent adverse event (AE) among patients with metastatic castration-resistant prostate cancer undergoing ADCs (58%; 95% CI: 40%–76%), fatigue (44%; 95% CI: 29%–59%), and nausea 40% (95 CI: 28%–53%). Above all, only three AEs ≥ grade III occurred at more than 10%. They were rash 10% (95% CI: 0%–28%) in urothelial cancer patients, thrombocytopenia 15% (95% CI: 9%–22%) and anemia 13% (95% CI: 7%–19%) respectively for renal cell carcinoma patients. Our results complement the recent study by Ren et al. on ADC monotherapies for urothelial carcinoma (Ren et al., 2024). While both studies share seven overlapping clinical trials, our analysis incorporated additional patient stratification and expanded the scope to include combination strategies with immunotherapy, ultimately encompassing 29 studies compared to their 12 monotherapy-focused investigations. Otherwise, we supplemented the analysis of efficacy with DOR to support the conclusion, which increased the reliability. These findings not only confirm the value of ADCs in urothelial carcinoma management but also highlight combination approaches as a promising therapeutic advancement with significant clinical potential.

While our analysis demonstrates promising clinical efficacy of ADCs in urological cancers, these benefits must be carefully balanced against their economic implications for real-world implementation. Currently, ADC therapies remain costly due to complex development and manufacturing processes, and their cost-effectiveness varies across healthcare systems (Zhu et al., 2022; Yeh et al., 2022; Wang et al., 2022). While improved response rates and survival outcomes may offset some of these expenses, broader accessibility will require pricing reforms, biosimilar competition, and value-based reimbursement models (Conti et al., 2021; Engelberg et al., 2022). Additionally, future studies should incorporate quality-of-life and cost-effectiveness analyses to better define their real-world utility. Despite these challenges, ADCs represent a significant therapeutic advance, and optimizing their affordability will be crucial for equitable patient access.

Our current meta-analysis has a number of limitations. First, a thorough investigation of the true effectiveness of ADCs in these advanced cancers was impeded by the small number of trials on patients with renal cell carcinoma, testicular cancer, and metastatic castration-resistant prostate cancer. Additionally, some subgroup analyses were not possible for these groups. More high-quality studies are needed to fully understand ADC potential in these cancers. It is important to note that our analysis included both early-phase studies (Phase I) and late-phase studies (Phase II and III), as well as patients at different stages, due to incomplete reporting of trial characteristics. Moreover, some included studies were terminated after Phase I, which may limit the clinical applicability of the pooled results. Therefore, future research should focus on conducting more robust, later-phase clinical trials in well-defined patient populations to confirm the efficacy and safety of ADCs in different urological cancer types. Also, our meta-analysis is limited by the significant heterogeneity observed across the studies for most of the investigated clinical indications. This heterogeneity is likely due to differences in study populations, treatment protocols, and outcome measures. The low sample sizes of many included studies further complicate the interpretation of the results. For instance, several studies included in our analysis had sample sizes of less than 50 patients, which limits the statistical power and precision of the effect estimates (Figure 2). These limitations make it challenging to draw definitive conclusions about the comparative clinical efficacy of different ADCs. Despite these challenges, our meta-analysis remains valuable for several reasons. First, it provides a comprehensive synthesis of the available evidence, highlighting the potential benefits of ADCs in treating urological cancers. Second, it identifies areas where further research is needed, particularly in renal cell carcinoma, testicular cancer, and metastatic castration-resistant prostate cancer. Future studies should aim to address these limitations by including larger sample sizes and using standardized outcome measures. Additionally, the use of random-effects models in our analysis helps account for the observed heterogeneity, providing a more conservative estimate of the treatment effects. Notably, with further clinical exploration of the use of ADCs in combination with immunotherapy, as well as the use of more types of ADCs in advanced urological cancers, it is believed that more promising therapeutic options will be available for patients in the near future.

## 5 Conclusion

In conclusion, the effectiveness and safety of administering ADCs to patients with advanced urological cancer, especially urothelial cancer, was fully validated by our meta-analysis. Meanwhile, renal cell carcinoma, testicular cancer, and metastatic castration-resistant prostate cancer all need further research to address these limitations by including larger sample sizes and using standardized outcome measures. It also warned us to be aware of several major adverse events related to various ADCs during the treatment of these cancers. The clinical data does have certain limitations, though, and further extensive multicenter randomized controlled studies are required to confirm our results.
