# Supplementary material for: Efficacy and safety of antibody-drug conjugates in the treatment of advanced urological cancers: a systematic review and a meta-analysis
Source: Front Pharmacol. 2025 Sep 1;16:1583654. doi: 10.3389/fphar.2025.1583654 (PMC12433865; doi:10.3389/fphar.2025.1583654)
Supplement: Supplementary file 1 [file Supplementaryfile1.docx]

**Supplementary materials**

Supplementary Table S1 Characteristics of the studies included in the meta-analysis.

| **Study** | **Year** | **NCT** | **Nation** | **Payload** | **Median follow up,month** | **Prior therapy** | **Metastasis** | | **Previous therapy lines** | |
| --- | --- | --- | --- | --- | --- | --- | --- | --- | --- | --- |
|  |  |  |  |  |  |  |  |  |  |  |
|  |  |  |  |  |  |  | **Yes** | **No** | **Number** | **Sample size** |
| Yuki Endo^36^ | 2024 | NR | Japan | MMAE | 8.20 (1.40-20.80) | Pembrolizumab Avelumab Taxane | 20 | 0 | NR | |
| Vadim S. Koshkin^14^ | 2022 | NR | America | MMAE | 7.2(3.7-11.6) | Chemotherapy  Radiation | 260 | 0 | 0 | 13 |
|  |  |  |  |  |  |  |  |  | 1 | 73 |
|  |  |  |  |  |  |  |  |  | 2 | 110 |
|  |  |  |  |  |  |  |  |  | ≥3 | 64 |
| AKINORI MINATO^37^ | 2024 | NR | Japan | MMAE | 8.0(5.2-14.5) | Chemotherapy+ Immunotherapy (Avelumab) | 20 | 60 | NR | |
|  |  |  |  |  |  | Chemotherapy+ Immunotherapy (Pembrolizumab) |  |  |  |  |
| Evan Y Yu^22^ | 2021 | NCT03219333 | America | MMAE | 13∙4 (11·3–18·9). | PD-1 or PD-L1 inhibitors | 88 | 1 | NR | |
| Jonathan E. Rosenberg^38^ | 2019 | NCT03219333 | America | MMAE | 10.2 (0.5-16.5) | PD-1 or PD-L1 inhibitors Platinum-based therapies Taxane | 125 | 0 | NR | |
| Peter H. O’Donnell^20^ | 2023 | NCT03288545 | America | MMAE | 15(12.7-17.4) | NR | 72 | 1 | NR | |
|  |  |  |  |  | 14.8(12.9-17.3) |  |  |  |  |  |
| Thomas Powles^23^ | 2021 | NCT03474107 | America | MMAE | 11.1 | PD-1 or PD-L1 inhibitor Platinum chemotherapy | NR | | NR | |
| Shunji Takahashi^21^ | 2019 | NCT03070990 | Japan | MMAE | NR | NR | NR | | NR | |
|  |  |  |  |  |  |  |  |  |  |  |
| Jonathan Rosenberg^39^ | 2020 | NCT02091999 | America | MMAE | NR | Platinum chemotherapy Taxanes PD-1 or PD-L1 inhibitors | 155 | | NR | |
| Thomas Powles^16^ | 2024 | NCT04223856 | America | MMAE | 17.2 | NR | NR | | 1and2 | 262 |
|  |  |  |  |  |  |  |  |  | ≥3 | 39 |
| Siming Li^40^ | 2023 | NCT04995419 | China | MMAE | NR | platinum-containing chemotherapy  PD-1 or PD-L1 inhibitors | NR | | NR | |
| Stefanie Zscha¨bitz^41^ | 2023 | NR | Germany | MMAE | NR | Chemotherapy PD-1 or PD-L1 inhibitors  FGFRinhibitor Sacituzumabgovitecan | 125 | | 1 | 1 |
|  |  |  |  |  |  |  |  |  | 2 | 68 |
|  |  |  |  |  |  |  |  |  | ≥3 | 18 |
| Makito Miyake^42^ | 2024 | NR | Japan | MMAE | NR | Platinum-based chemotherapy Immune checkpoint inhibitors | NR | | NR | |
| Wataru Fukuokaya^43^ | 2024 | NR | Japan | MMAE | 8.9 (0.1–16.4) | NR | NR | | NR | |
| AKINORI MINATO^44^ | 2023 | NR | Japan | NR | 7.5 (4.9-10.8) | platinum-based chemotherapy andimmunotherapy (avelumab or pembrolizumab) | 26 | 0 | 2 | 18 |
|  |  |  |  |  |  |  |  |  | ≥3 | 8 |
| Scott T. Tagawa^45^ | 2021 | NCT03547973 | America | SN-38 | 9.1 (0-19.9) | Platinum anticancer therapy  Immune checkpoint inhibitor Enfortumab vedotin  Erdafitinib | NR | | 1 | 22 |
|  |  |  |  |  |  |  |  |  | 2 | 30 |
|  |  |  |  |  |  |  |  |  | ≥3 | 56 |
| Petros Grivas^46^ | 2024 | NCT03547973 | America | SN-38 | 14.8 (12.6-16.8) | Platinum-based chemotherapy Immune checkpoint inhibitor | 41 | 0 | NR | |
| Daniel P. Petrylak^24^ | 2024 | NCT03547973 | America | SN-38 | 9.3 (0.5-30.6) | Immune checkpoint inhibitor | 38 | | NR | |
| B. A. McGregor^47^ | 2024 | NCT04724018 | America | MMAE/SN-38 | 14 | Immunotherapy  Cisplatin-based chemotherapy Carboplatin-base chemotherapy | 23 | 0 | 1 | 1 |
|  |  |  |  |  |  |  |  |  | 2 | 11 |
|  |  |  |  |  |  |  |  |  | ≥3 | 11 |
| A. Bardia^25^ | 2021 | NCT01631552 | America | SN-38 | 8.97 ( 0.26 55.72) | NR | 45 | | NR | |
| Yongbao Wei^48^ | 2023 | NR | China | MMAE | 12(8-17) | Diagnostic-TURBT GC + BCG Right-sided laparoscopic radical resection GC + Triprilimab | NR |  | NR | |
| Xinan Sheng^49^ | 2024 | NCT03507166 | China | MMAE | 19.6 | Cisplatin-containing chemotherapy PD-1/PD-L1 therapy Paclitaxel-containing chemotherapy | 40 | 3 | 1 | 29 |
|  |  |  |  |  |  |  |  |  | ≥2 | 14 |
|  |  | NCT03809013 | China | MMAE | 23.4 |  | 64 | 0 | 1 | 9 |
|  |  |  |  |  |  |  |  |  | ≥2 | 55 |
| Huayan Xu^26^ | 2022 | NCT04073602 | China | MMAE | NR | NR | 13 | 6 | 1 | 4 |
|  |  |  |  |  |  |  |  |  | ≥2 | 15 |
| Xinan Sheng^50^ | 2023 | NCT04264936 | China | MMAE | NR | NR | 22 | 19 | 0 | 25 |
|  |  |  |  |  |  |  |  |  | ≥1 | 16 |
| Jingwei Xu^27^ | 2023 | NR | China | MMAE | 10.6 (2.2-19.4) | NR | NR | | NR | |
|  |  |  |  |  |  |  |  |  |  |  |
| Meiting Chen^51^ | 2023 | NR | China | MMAE | NR | Platinum-based chemotherapy PD-1/PD-L1 therapy Enfortumab vedotin | NR | | NR | |
| Wasilijiang^52^ | 2024 | NCT06178601 | China | MMAE | NR | NR | 10 | 13 | NR | |
| Kejia Zhu^53^ | 2024 | NR | China | MMAE | 14.0 (1-19) | NR | NR | | NR | |
|  |  |  |  |  |  |  |  |  |  |  |
| Jinchao Chen^54^ | 2024 | NR | China | MMAE | 9.0 (2-46) | Chemotherapy Immunotherapy | 103 | 0 | 1 | 40 |
|  |  |  |  |  |  |  |  |  | 2 | 41 |
|  |  |  |  |  |  |  |  |  | ≥3 | 22 |
| John A. Thompson^55^ | 2018 | NCT01114230 | America | MMAE | NR | Anti angiogenic therapy | 26 | 0 | NR | |
|  |  | NCT01672775 | America |  |  |  |  |  |  |  |
| Bradley A. McGregor^56^ | 2020 | NCT02837991 | America | MMAE | 15.2 (12.9-17.6) | Immune checkpoint inhibitor VEGF/VEGFR-directed therapy MTOR inhibitor | NR | | NR | |
| Christian Kollmannsberger^13^ | 2021 | NCT02639182 | Canada | MMAF | NR | NR | 67 | 0 | NR | |
| Christophe Massard^57^ | 2019 | NCT01497821 | France | DM1 | NR | NR | 37 | 0 | NR | |
| Sumanta K. Pal^58^ | 2019 | NCT02216890 | America | PBD | NR | NR | 18 | 0 | NR | |
| Andrea Necchi^59^ | 2016 | NCT01851200 | Italy | MMAE | NR | NR | NR | | NR | |
| Ryan Ashkar^60^ | 2021 | NCT01461538 | America | MMAE | NR | NR | NR | | NR | |
| Deaglan McHugh^61^ | 2019 | NCT01228760 | America | SLC44A | NR | NR | 46 | | NR | |
| Matthew I. Milowsky^15^ | 2016 | NCT00070837 | America | PSMA | NR | Surgery Radiation Chemotherapy Hormone therapy | 62 | | NR | |
| Matthew D. Galsky^62^ | 2008 | NCT00052000 | America | PSMA | NR | NR | 23 | | NR | |
| Johann S.De Bono^63^ | 2021 | NCT02991911 | UK | PSMA | 5.9 (1.7–25.3) | Radiotherapy Surgery Abiraterone Enzalutamide | 33 | | NR | |
| Daniel P. Petrylak^64^ | 2019 | NCT01414283 | America | PSMA | NR | chemotherapy regimens | 52 | | NR | |
| Daniel P. Petrylak^65^ | 2020 | NCT01695044 | America | PSMA | NR | Chemotherapy-experienced Chemotherapy-naïve | 119 | | NR | |
| [Results Posted on ClinicalTrials.gov](https://clinicaltrials.gov/study/NCT02020135?tab=results&a=1)^66^ | NR | NCT02020135 | America | PSMA | NR | Chemotherapy-experienced Chemotherapy-naïve | 9 | | NR |  |
| J. Shen^67^ | 2023 | NCT04662580 | America | PSMA | NR | NR | 24 | | NR | |
| Daniel C. Danila^68^ | 2019 | NCT01283373 | America | STEAP1 | NR | Docetaxel Cabazitaxel Abiraterone Enzalutamide | 77 | | NR | |
| Rahul Raj Aggarwal^69^ | 2022 | NCT03575819 | America | CD46 | NR | NR | 33 | | NR | |


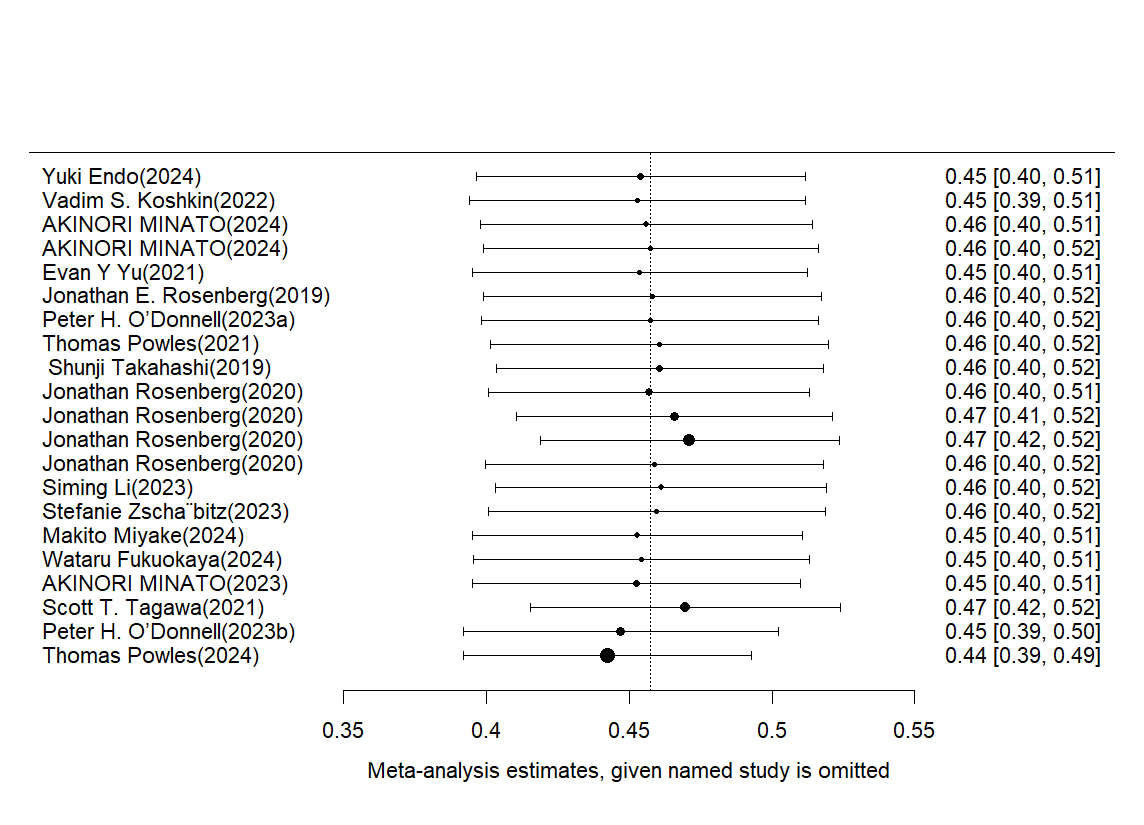


**b**


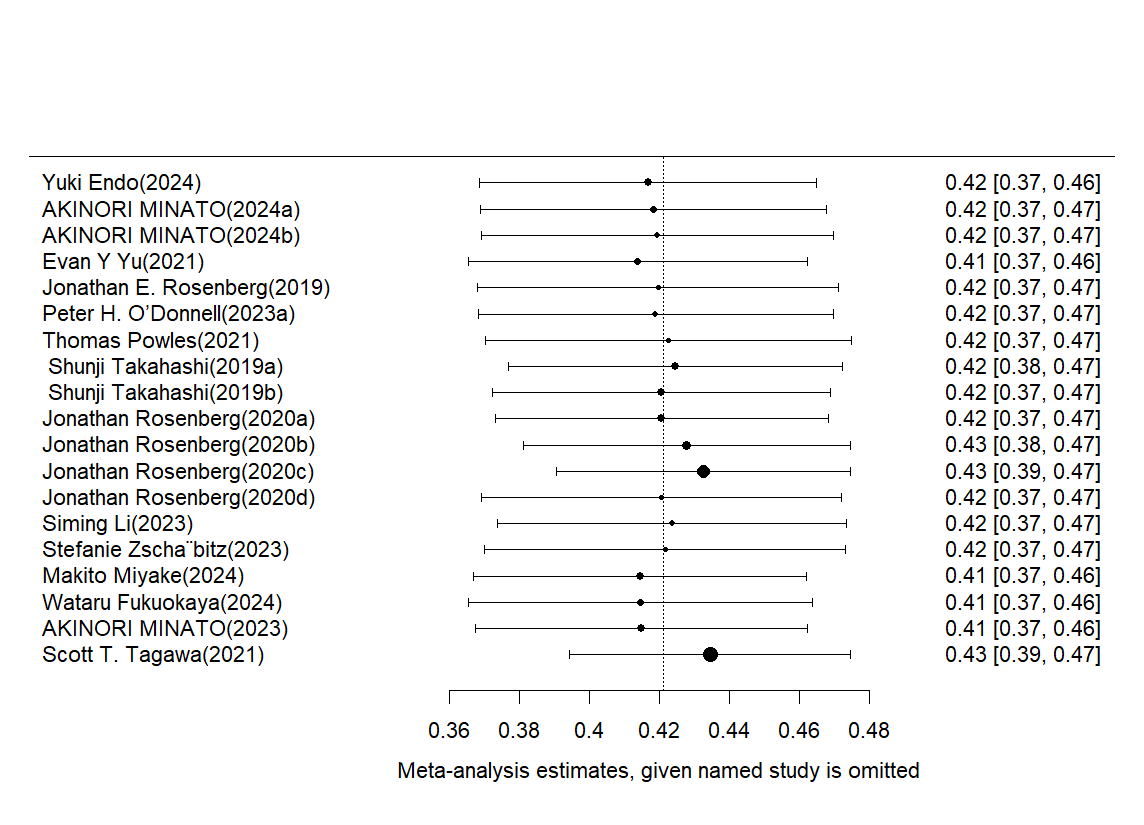


**c**


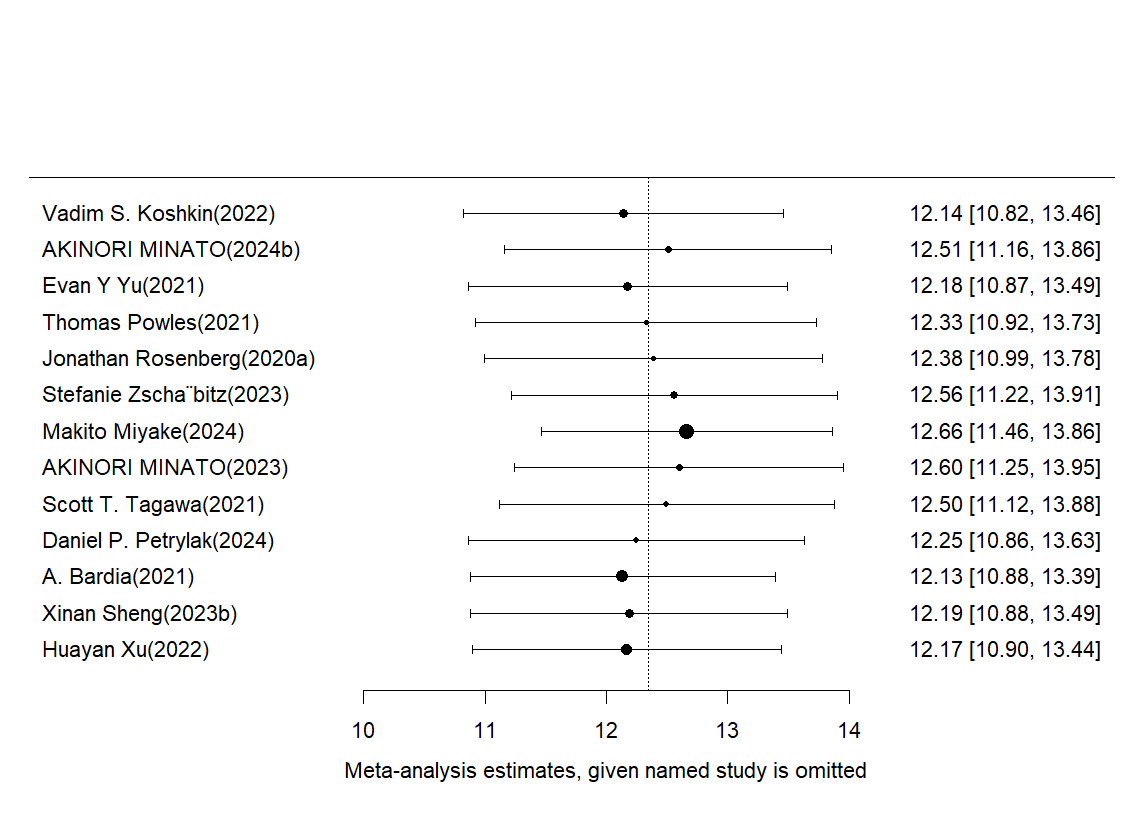


**d**


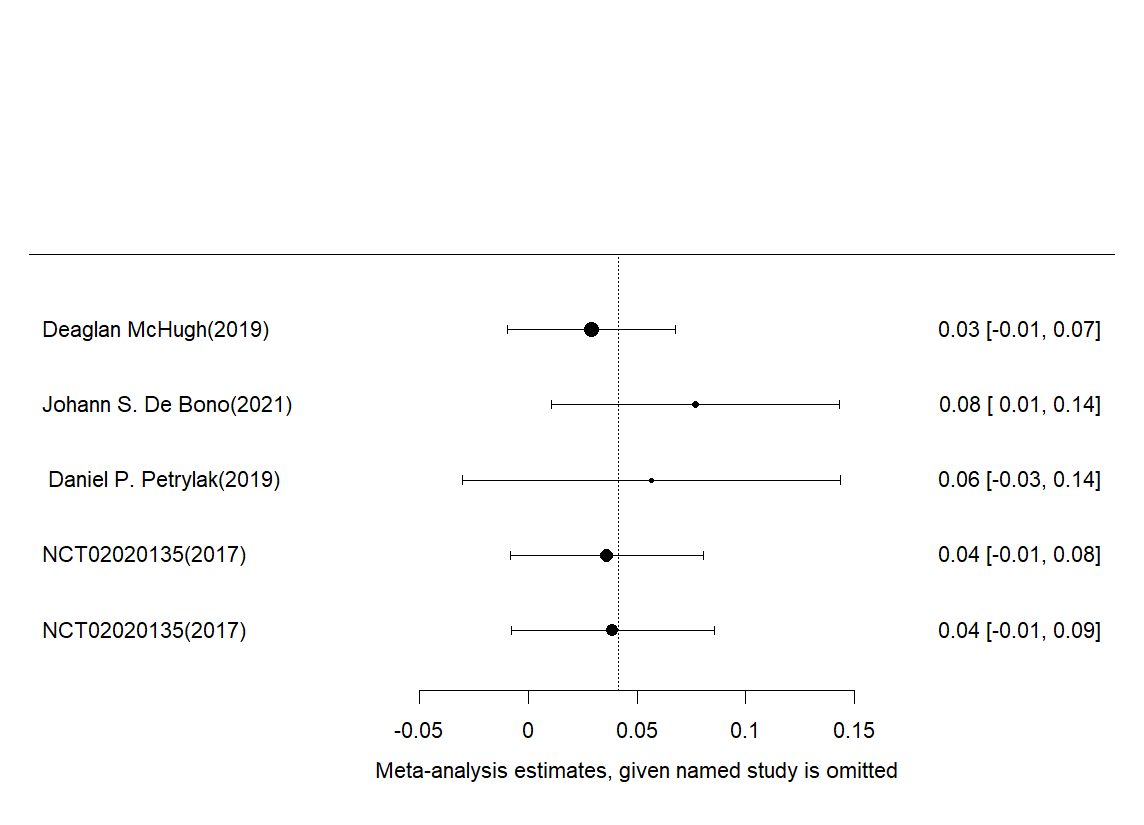


**e**


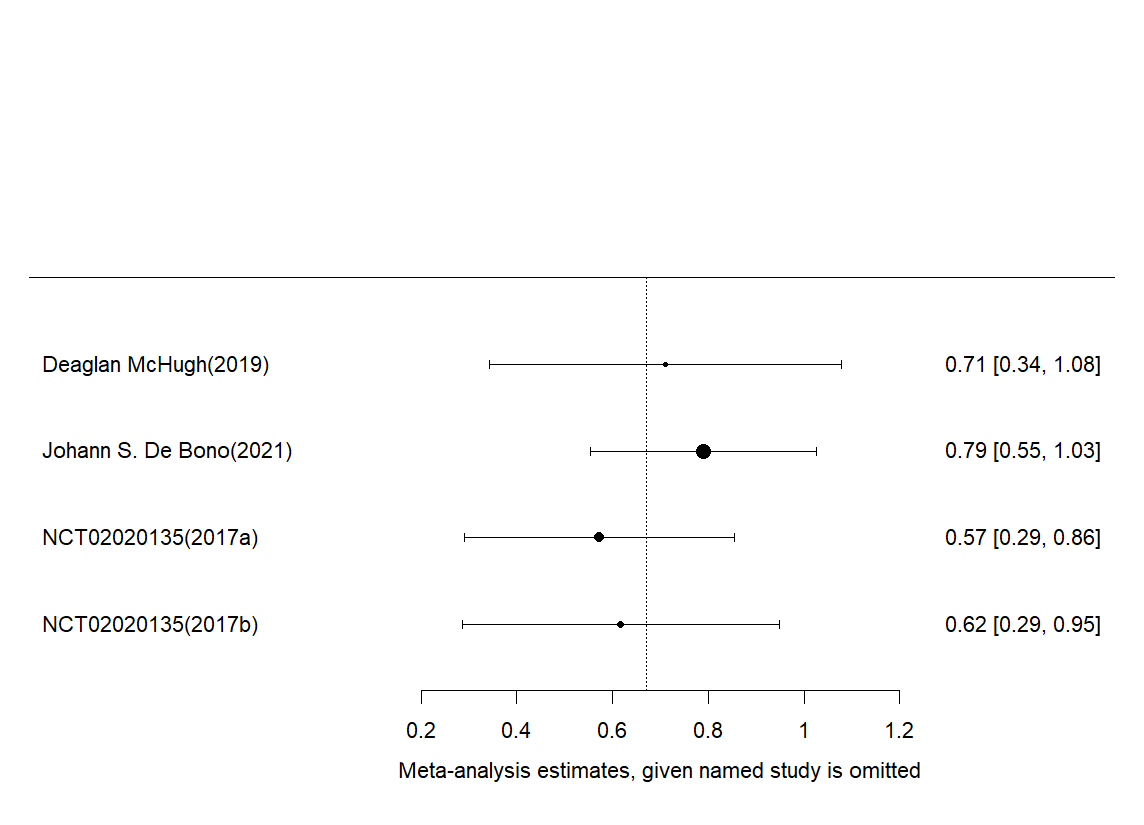


**f**


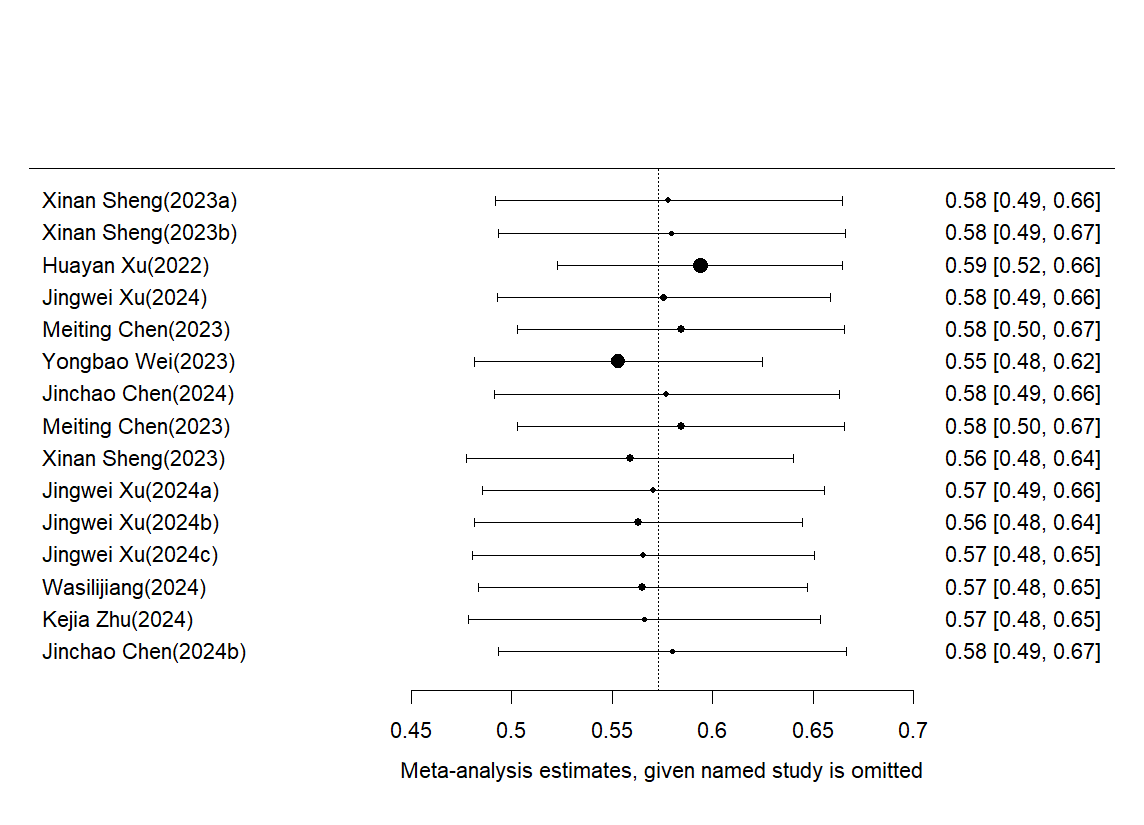


**a**

Supplementary Figure S1 Sensitivity analysis. (a) Sensitivity analysis for ORR in DV monotherapy group and DV combination therapy with pembrolizumab group of urothelial cancer studies, (b) Sensitivity analysis for ORR in EV monotherapy group and EV combination therapy with pembrolizumab group of urothelial cancer studies, (c) Sensitivity analysis for ORR in 1.25mg/kg EV intervention group and non-1.25mg/kg EV intervention group of urothelial cancer studies, (d) Sensitivity analysis for OS in all urothelial cancer studies, (e) Sensitivity analysis for ORR in all metastatic castration-resistant prostate cancer studies. (f) Sensitivity analysis for DCR in all metastatic castration-resistant prostate cancer studies. ORR, objective response rate; OS, overall survival; DCR, disease control rate; DV, disitamab vedotin; EV, enfortumab vedotin.

Supplementary Figure S2 Publication bias. (a) Publication bias for ORR in DV monotherapy group and DV combination therapy with pembrolizumab group of urothelial cancer studies, (b) Publication bias for ORR in EV monotherapy group and EV combination therapy with pembrolizumab group of urothelial cancer studies, (c) Publication bias for ORR in 1.25mg/kg EV intervention group and non-1.25mg/kg EV intervention group of urothelial cancer studies, (d) Publication bias for PFS in all urothelial cancer studies, (e) Publication bias for ORR in all Renal cell cacinoma studies. (f) Publication bias for DCR in all metastatic castration-resistant prostate cancer studies. ORR, objective response rate; PFS, progression free survival; DCR, disease control rate; DV, disitamab vedotin; EV, enfortumab vedotin.


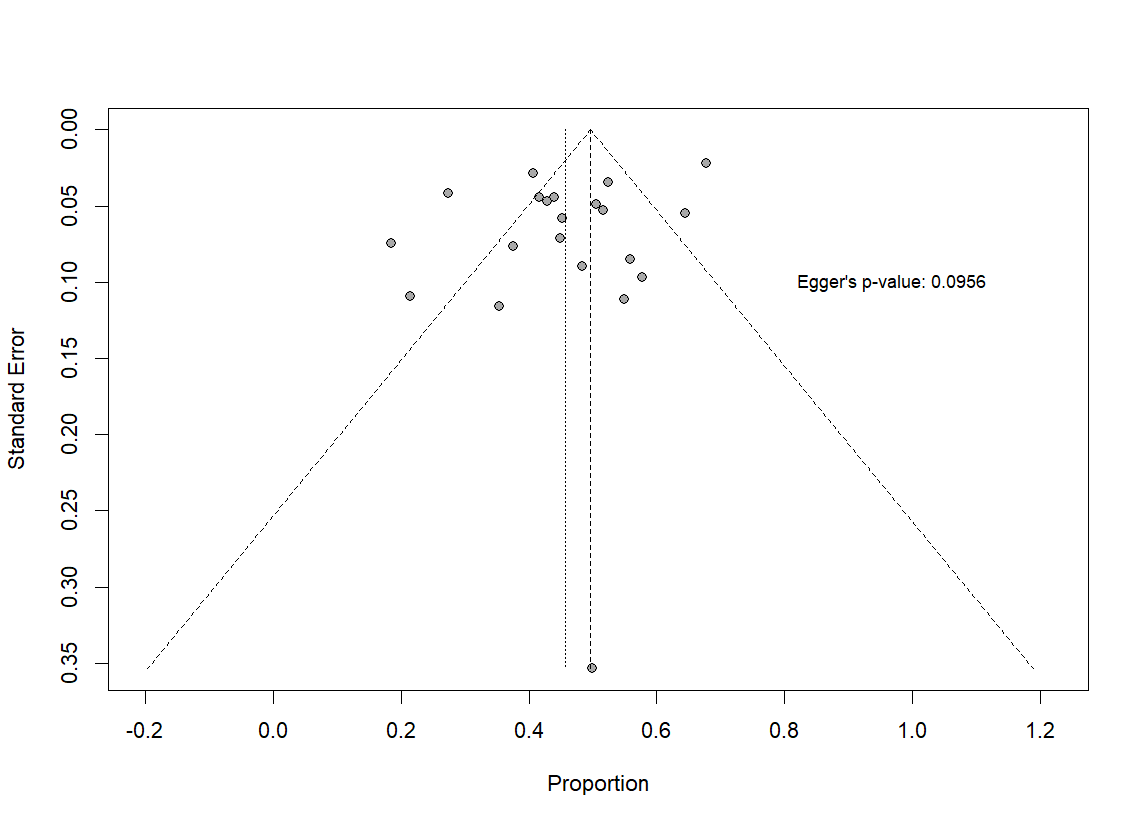


**b**


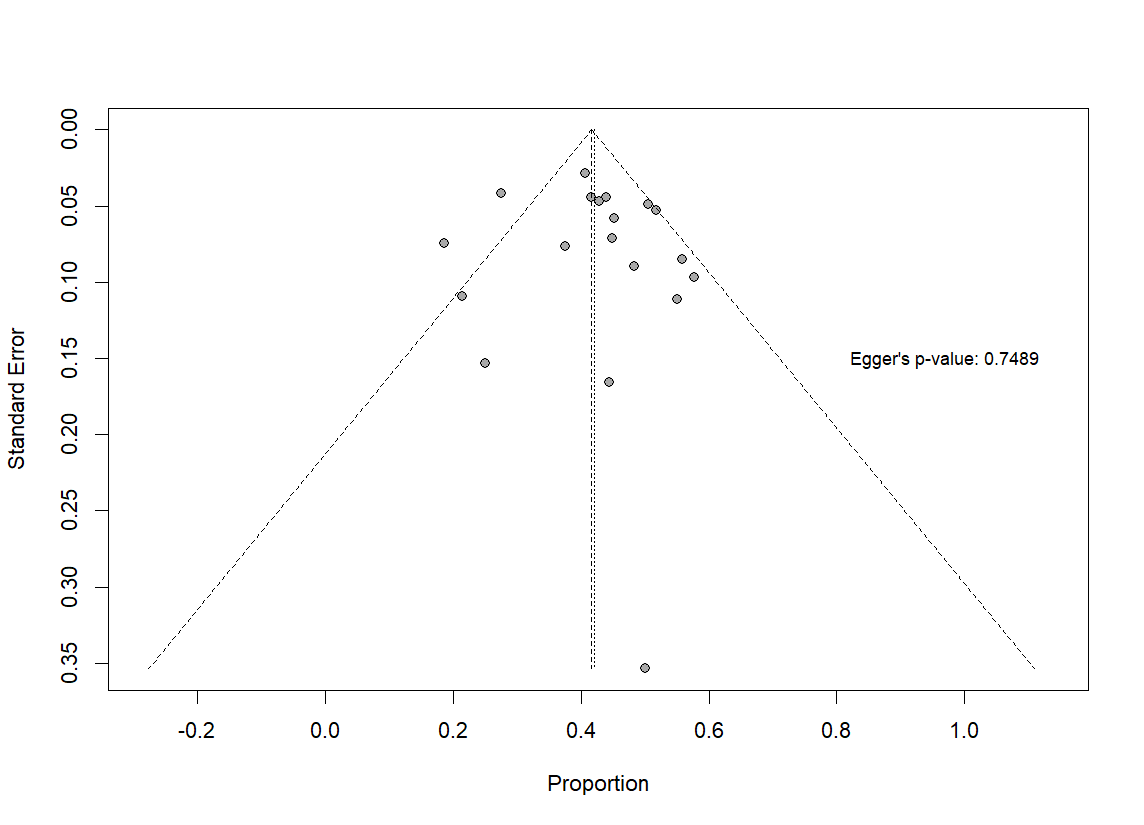


**c**


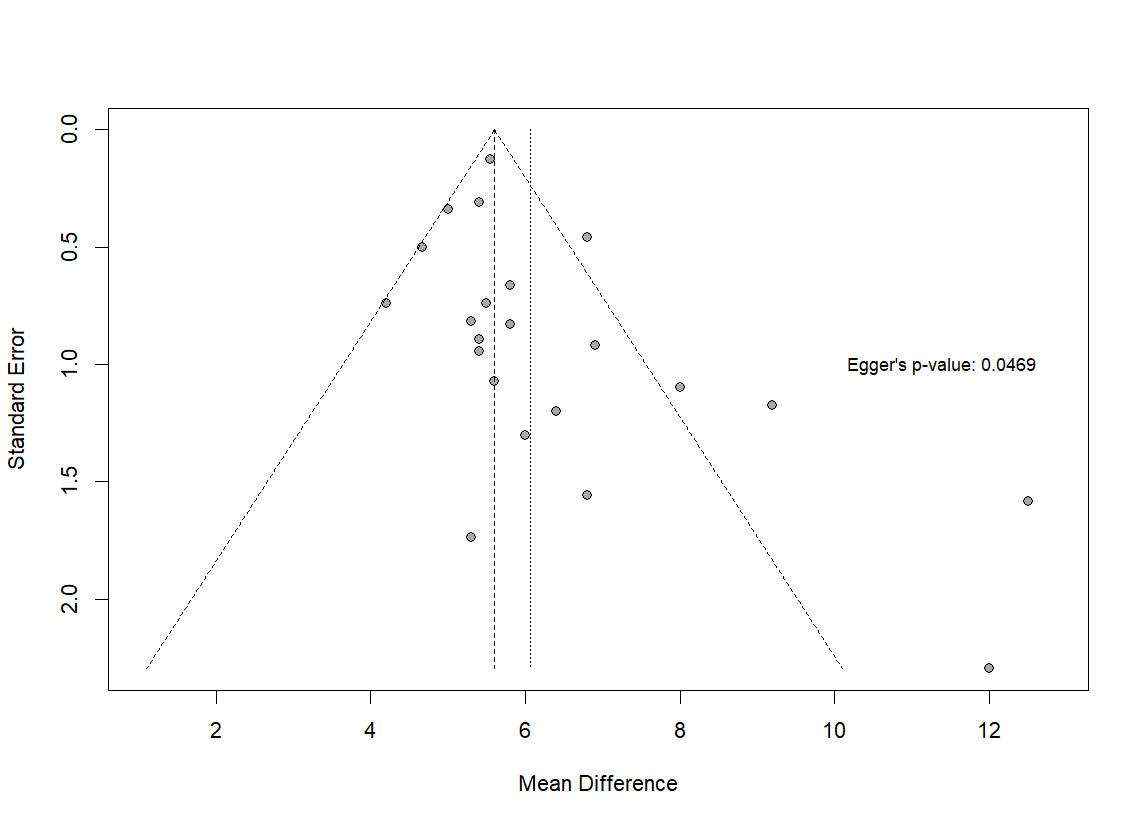


**d**


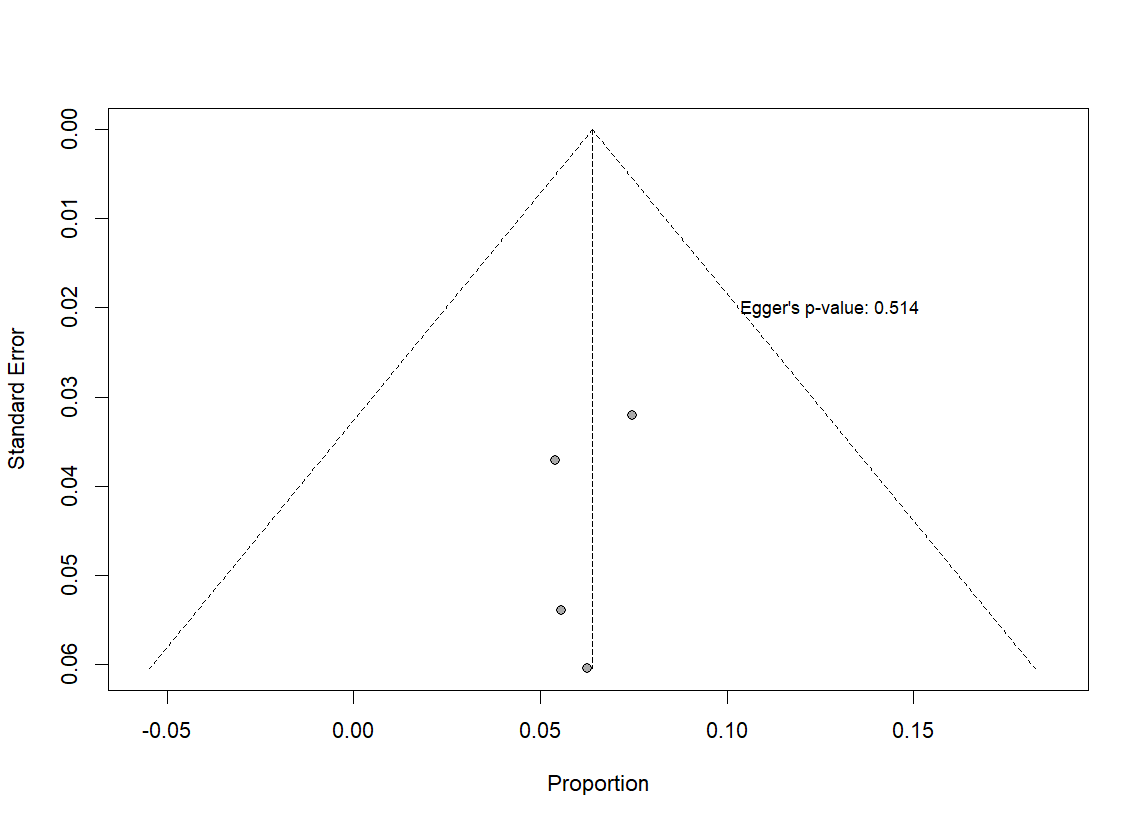


**e**


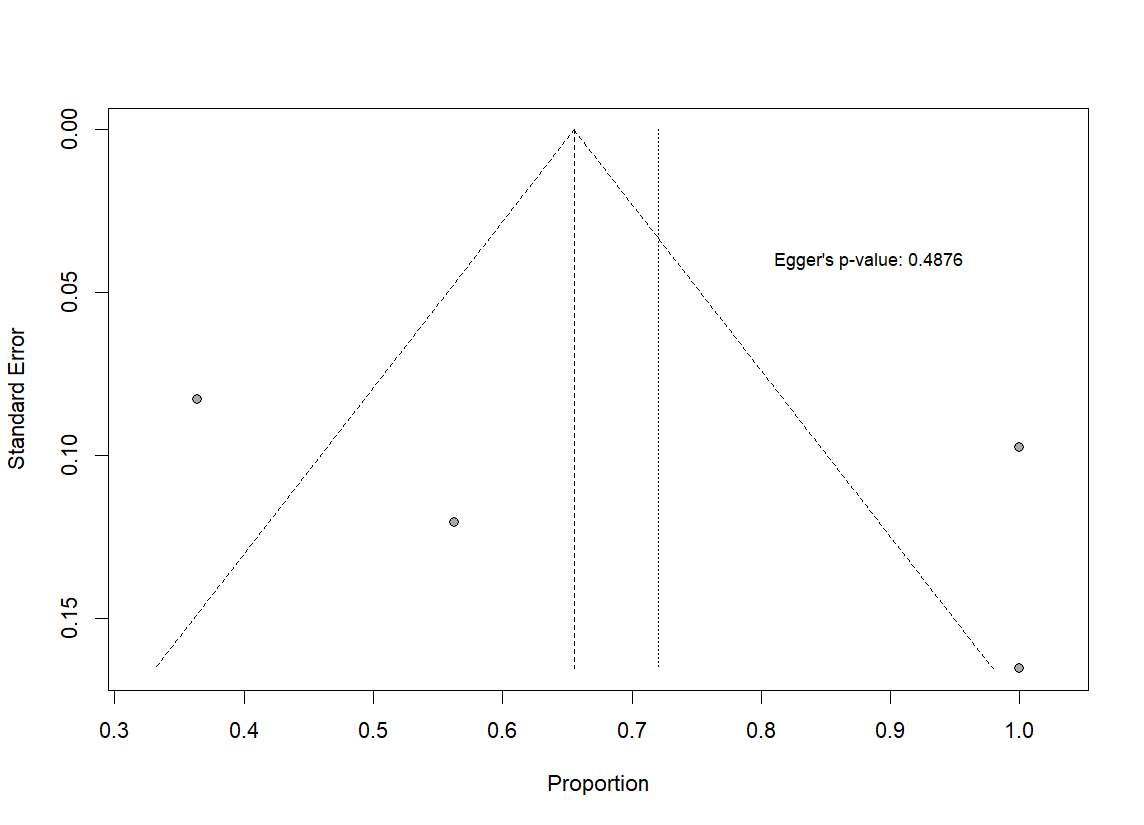


**f**


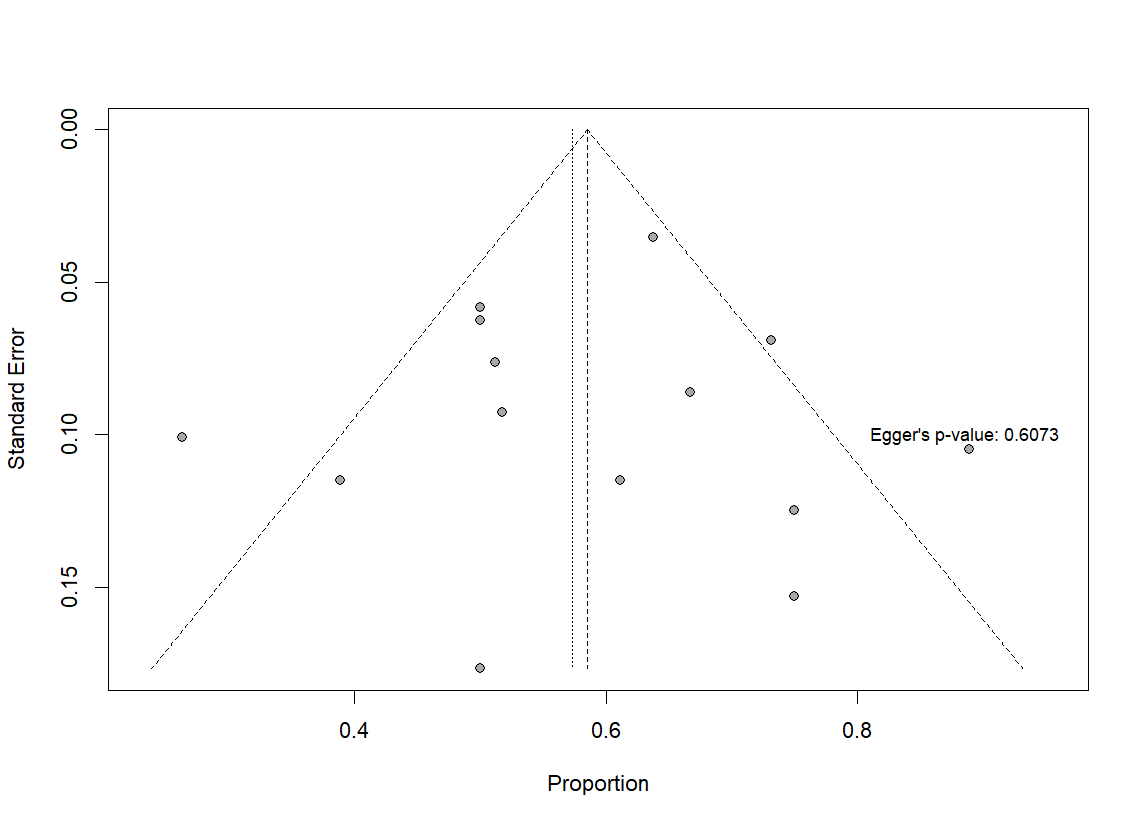


**a**
